# Molecular Epidemiology and Phylogenetic Analyses of Influenza B Virus in Thailand during 2010 to 2014

**DOI:** 10.1371/journal.pone.0116302

**Published:** 2015-01-20

**Authors:** Nipaporn Tewawong, Kamol Suwannakarn, Slinporn Prachayangprecha, Sumeth Korkong, Preeyaporn Vichiwattana, Sompong Vongpunsawad, Yong Poovorawan

**Affiliations:** Center of Excellence in Clinical Virology, Faculty of Medicine, Chulalongkorn University, Bangkok, Thailand; University of Maryland, UNITED STATES

## Abstract

Influenza B virus remains a major contributor to the seasonal influenza outbreak and its prevalence has increased worldwide. We investigated the epidemiology and analyzed the full genome sequences of influenza B virus strains in Thailand between 2010 and 2014. Samples from the upper respiratory tract were collected from patients diagnosed with influenza like-illness. All samples were screened for influenza A/B viruses by one-step multiplex real-time RT-PCR. The whole genome of 53 influenza B isolates were amplified, sequenced, and analyzed. From 14,418 respiratory samples collected during 2010 to 2014, a total of 3,050 tested positive for influenza virus. Approximately 3.27% (471/14,418) were influenza B virus samples. Fifty three isolates of influenza B virus were randomly chosen for detailed whole genome analysis. Phylogenetic analysis of the HA gene showed clusters in Victoria clades 1A, 1B, 3, 5 and Yamagata clades 2 and 3. Both B/Victoria and B/Yamagata lineages were found to co-circulate during this time. The NA sequences of all isolates belonged to lineage II and consisted of viruses from both HA Victoria and Yamagata lineages, reflecting possible reassortment of the HA and NA genes. No significant changes were seen in the NA protein. The phylogenetic trees generated through the analysis of the PB1 and PB2 genes closely resembled that of the HA gene, while trees generated from the analysis of the PA, NP, and M genes showed similar topology. The NS gene exhibited the pattern of genetic reassortment distinct from those of the PA, NP or M genes. Thus, antigenic drift and genetic reassortment among the influenza B virus strains were observed in the isolates examined. Our findings indicate that the co-circulation of two distinct lineages of influenza B viruses and the limitation of cross-protection of the current vaccine formulation provide support for quadrivalent influenza vaccine in this region.

## Introduction

Influenza virus belongs to the *Orthomyxoviridae* family of enveloped, segmented negative-stranded RNA viruses. Influenza A and B viruses are major causes of respiratory infection in human and contribute to increasing morbidity and mortality globally [1]. Influenza A virus infects humans, swines, birds, and horses, whereas influenza B virus infects humans and seals [2]. There are 18 subtypes of influenza A virus, of which H1, H2, and H3 are known to infect humans, while other subtypes such as H5, H6, H7 and H9 have the potential to cause human pandemics [3]. In contrast, influenza B virus has no subtypes.

The first isolated strain of influenza B virus was B/Lee/40 [4]. Since 1983, influenza B viruses evolved antigenically and genetically into two major lineages: B/Victoria/2/87-like and B/Yamagata/16/88-like [5]. Currently, Victoria and Yamagata lineages have continually co-circulated in many regions of the world [6]. Although the trivalent seasonal influenza vaccines include one strain of influenza B virus, evidence suggests that the current vaccines can be improved by including both lineages [7].

The genome of influenza B consists of eight segments: polymerase basic-1 (PB1), PB2, polymerase acidic (PA), haemagglutinin (HA), nucleoprotein (NP), neuraminidase (NA), matrix (M), and nonstructural protein (NS). Binding of the virus to its cellular receptors, terminal sialic acids of glycoproteins and glycolipids, is mediated by the viral surface glycoprotein HA [8]. HA forms a homotrimer with each monomer composed of an HA1 and HA2 subunit. HA1 is the receptor-binding subunit of HA and represents the major antigenic sites that undergo constant antigenic variations due to frequent amino acid substitutions and insertion/deletions [9]. In contrast, the hydrophobic N-terminus of HA2 is the fusion peptide, which is the most conserved and play role for inducing fusion of viral envelope and endosomal host membrane [10].

Genetic reassortment is a crucial process of evolution for segmented RNA viruses, including influenza B virus, which effectively generates new recombinant genome most fit for viral adaptation [11,12]. Previous studies revealed that the rates of antigenic drift and evolution of influenza type B viruses are lower than in influenza type A [13]. Influenza A viruses are able to undergo antigenic shift by genetic reassortment between different subtypes [14], while influenza B viruses resort to various mechanism of deletion, insertion, and substitution within different co-circulating strains [9]. This antigenic drift allows influenza B virus to escape host immunity and continue to adapt/evolve without antigenic shift [9,11,15], thus explaining the limited virus diversity and pandemic potential [15,16].

Although the Ministry of Public Health in Thailand encouraged individuals ≥65 years, those with underlying medical conditions (asthma, heart diseases, diabetes, etc.), and pregnant women to receive yearly influenza vaccination, universal vaccination is not implemented and vaccination coverage is relatively low. Data on individuals seeking vaccination from private healthcare facilities are lacking. As a result, the rising prevalence and increasingly severe cases of influenza B virus infection have been reported [17–19]. A study of prevalence and epidemiological data among influenza B virus in Thailand was previously described [20], but molecular characterization and genetic evolutionary profiles of influenza B virus in Thailand remained unclear. In this study, we determined the prevalence of influenza B virus infection in Thailand from individuals with influenza-like illness during January 2010 to February 2014. We also characterized the gene segments of influenza B virus on the basis of genetic clustering, phylogenetic topology and pairwise amino acid variations.

## Materials and Methods

### Study population and sample collection

From January 2010 to February 2014, a total of 14,418 upper respiratory specimens from patients with influenza-like illness were collected from Bangkok, Khon Kaen and Surat Thani provinces in Thailand. The inclusion criteria were fever (> 38°C) combined with respiratory symptoms such as cough, sore throat and runny nose. The specimens were collected in the viral transport medium and sent to the Center of Excellence in Clinical Virology, Faculty Medicine, Chulalongkorn University for testing of respiratory viruses. All samples were stored at -70°C.

### Ethical consideration

This research was performed on respiratory specimens stored as anonymous. All patient identifiers were removed to protect patient confidentiality and no personal information appeared in any part of the document in this study. The Institutional Review Board of the Faculty of Medicine at Chulalongkorn University approved the research protocol (IRB number 337/57). The IRB waived the need for consent because the samples were anonymous.

### Influenza B screening by one-step multiplex real-time RT-PCR

Viral RNA was extracted from samples by using a commercially available Viral Nucleic Acid Extraction Kit (RBC Bioscience Co, Taiwan) following the manufacturer’s instruction. Influenza virus detection was performed with one-step multiplex real-time RT-PCR assays based on TaqMan probes as previously described [21–23]. In addition, the GAPDH gene served as an internal control, while the matrix (M) gene of influenza A and B was amplified to characterize the types of influenza virus. In brief, the 15 μl reaction volume contained 7.5 μl of 2X reaction buffer (included dNTPs), 1.825 μl of RNAse-free H_2_0, 0.3 μl of Superscript III enzyme mix (Taq DNA polymerase and reverse transcriptase; Invitrogen), 0.375 μl of 10 nmol/L each reverse and forward primer, and 2 μl of template RNA. Amplification was performed on Rotor-Gene3000 (Corbett Research, New South Wales, Australia) with a single reverse transcription step of 50°C for 45 min, “hot start PCR” at 95°C for 2 minutes, followed by 50 amplification cycles of denaturation for 30 seconds, primer annealing at 55°C for 10 seconds, extension at 60°C for 10 seconds, and a final extension step at 72°C for 20 seconds.

### Conventional PCR and sequencing

One sample positive for influenza B virus was randomly chosen each month for whole genome analysis. Viral cDNA was synthesized using the M-MLV reverse-transcription system (Promega, Madison, WI) and 1 mM universal primers as described [22]. The whole genome sequences were amplified by primer sets for influenza B virus (S1 Table). Briefly, the reaction volume contained 10 ml of 2.5 X Eppendorf mastermix (5Prime, Hamburg, Germany), 0.25 mM MgCl_2_, 0.5 mM forward and reverse primers, 2 μl of cDNA template and RNAse-free H_2_0 to the final volume of 25 μl. Amplification was carried out in a thermal cycler (Eppendorf, Germany) under the following conditions: initial denaturation at 94°C for 3 minutes, 40 cycles of 30 seconds denaturation at 94°C, 30 seconds of primer annealing at 50°C (for PB2, PB1, and PA genes) and 55°C (for HA, NP, NA, MP, and NS genes), 90 seconds of extension at 72°C, and further extension for 7 minutes at 72°C. PCR products were separated on a 2% agarose gel with a 100-bp DNA ladder and visualized on a UV trans-illuminator. PCR products were gel-purified using the HiYield Gel DNA Fragment Extraction kit (RBC Bioscience Co, Taiwan). DNA sequencing was performed by First BASE Laboratories Sdn Bhd (Selangor, Malaysia).

**Phylogenetic analysis**. SeqMan II program of the DNAStar software (v6.0) was used for nucleotide sequence assembly. Genome sequences were aligned using ClustalW implemented in the BioEdit program (v7.2.0). MEGA program (v6.06) was used for the phylogenetic tree construction by applying the neighbor-joining method with Kimura’s two-parameter distance model and 1,000 bootstrap replicates. Sequences representative from different areas of the world available in GenBank and GISAID databases and those of Southern hemisphere vaccine strains recommended by WHO for the influenza seasons from 2006 to 2014 (S2 Table) were included in phylogenetic analysis. The latter virus sequences were also used as references to compare the amino acid substitutions with influenza B Thailand strains. The relative amino acid frequency in the analysis of the genome signatures for each gene was done using WebLogo [24].

### Meteorological data

Thailand is located in a tropical area between latitudes 5° 37′N to 20°27′N and longitudes 97° 22′E to 105° 37′E. Its climate is characterized by the rainy season (mid-May to mid-October) due to the southwest monsoon, winter season (mid-October to mid-February) due to the northeasterly wind, and hot dry summer or pre-monsoon season (mid-February to mid-May) [25].

### Statistical analysis

Statistical data analysis was carried out using the Statistical Package for Social Sciences version 19.0 (SPSS Inc., Chicago, USA). The Chi-square test was used to analyze demographic patient factors. All data were considered statistically significant at a *p*-value less than 0.05.

### Accession numbers

The whole genome sequences of the influenza B isolates in Thailand during 2012 to 2014 are available in GenBank (Accession numbers KM100190-KM100333). The complete gene sequences of all strains in 2010 to 2011 have been previously deposited in GenBank (Accession numbers JX512971-JX513210).

## Results

### Demographic profile and seasonality

**Figure 1:**
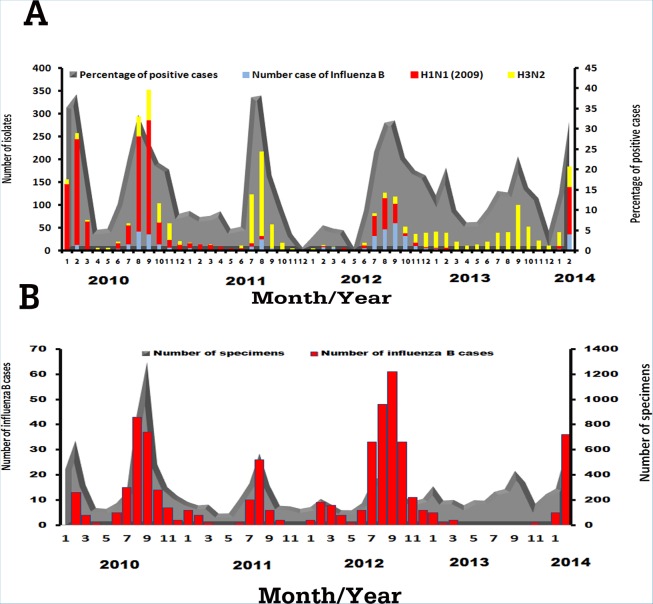
Incidence of influenza A and B viruses identified from clinical samples between 2010 and 2014. (A) The distribution of the influenza isolates for each month, including influenza B (light blue color), influenza A(H1N1)pdm09 (red color), and influenza A(H3N2) (yellow color) shown as bars (left scale). From the total number of specimens collected every month, the percent of influenza-positive cases are shown as grey color area under the curve (right scale). (B) Bar graph illustrating only the total number of influenza B infection monthly (left scale) relative to the number of total specimens collected each month are shown in gray (right scale).

Among 14,418 patients with influenza-like illness, a total of 3050 patient samples tested positive for influenza viruses. We found 1387 samples positive for influenza A H1N1 pdm09 (45.5%), 1192 for influenza A H3N2 (39.1%), and 471 for influenza B (15.4%). Seasonality of each virus varied from year to year (Fig. 1A). From 2010, influenza A H1N1 pdm09 was the prevalent subtype among influenza-positive samples. In the second half of 2011 and most of 2013, influenza A H3N2 virus predominated.

Influenza B, however, was detected yearly between 2010 and 2014. Overall, influenza B infection accounted for approximately 3.2% of all patient samples. Approximately 47.8% with influenza B were male and 52.2% were female (1:1.09 ratio), which was not a statistically significant difference (p = 0.725) (Table 1). For individuals in which age information was available, we stratified them into five different age groups. Those between 5–19 years represented 22.7% of all patients, but they constituted the majority (41.2%) of all influenza B infection (p < 0.0001). Specimens tested positive for influenza B virus were nasal (73.5%), nasopharyngeal (18.3%), and throat (8.3%) swabs. The majority of influenza B cases were collected in Bangkok (89.4%).

**Table 1:** Demographic characteristics of patients (N = 14,418).

**Parameter**	**Variable**	**No. specimens (%)**	**No. patients (%)**	**% positive rate^a^**	***P* value^b^**
**Gender**	Male	7002 (48.6)	225 (47.8)	3.22	0.725
	Female	7416 (51.4)	246 (52.2)	3.32	
**Age (Years)**	< 5	4029 (27.9)	46 (9.7)	1.15	
	5–19	3280 (22.7)	194 (41.2)	5.92	<0.0001
	20–44	2713 (18.8)	99 (21.0)	3.65	
	45–64	993 (6.8)	39 (8.3)	3.93	
	> 65	429 (2.9)	7 (1.5)	1.64	
	N/A	2974	86		
**Type of specimens**	Nasopharyngeal swab	2275 (15.8)	86 (18.3)	3.78	
	Nasopharyngeal aspirate	5 (0.1)	0	0	
	Nasal swab	7475 (51.8)	346 (73.5)	4.63	<0.0001
	Throat swab	4663 (32.3)	39 (8.3)	0.84	
**Provinces**	Bangkok	8916 (61.8)	421 (89.4)	4.73	<0.0001
	Khon Kaen	4652 (32.3)	39 (8.3)	0.84	
	Surat Thani	850 (5.9)	11 (2.3)	1.3	

^a^ Calculated by (number of patients/number of specimen) x 100.

^b^ At least one of the expected values is smaller than 0.05, the *P* value is calculated by the Chi square test.

N/A Information not available.

Most influenza B occurred in 2012, while fewest cases were found in 2013 (Fig. 1B). From 2010 to 2012, influenza B generally peaked between July and September and coincided with the local rainy season. The annual incidence of influenza B compared to all influenza-positive cases was 2.6% in 2010 (141/5326), 2.2% in 2011 (56/2545), 8.3% in 2012 (222/2676), 0.37% in 2013 (11/3000), and 4.71% in the first two months of 2014 (41/871).

### Sequence and phylogenetic analysis

**Figure 2:**
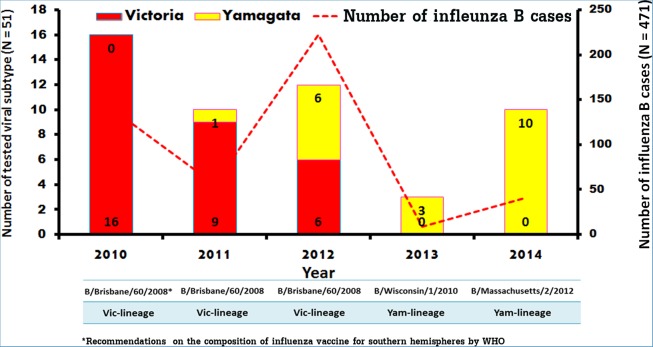
Analysis of the numbers and influenza B strains of randomly sampled sequences from January 2010 to February 2014. Number of B/Victoria and B/Yamagata lineage strains found are displayed in red and yellow bars, respectively (left scale). Total number of influenza B positive samples for each year is indicated by dot-line (right scale). The strains B/Brisbane/60/2008, B/Wisconsin/1/2010, and B/Massachusetts/2/2012 included in the Southern hemisphere vaccines for the given year are indicated by the asterisk. Vic denotes Victoria and Yam denotes Yamagata.

To identify the lineage of the influenza B virus circulating in Thailand during these years, 51 samples which tested positive for influenza B virus by real-time RT-PCR were randomly selected (at least one isolate per month when available) and the entire haemagglutinin (HA) gene was sequenced. Evaluation of the HA nucleotide sequences revealed that all influenza B isolates from 2010 belonged to the B/Victoria lineage (Fig. 2). Interestingly, co-circulation of influenza B/Victoria lineage (68.2%) and B/Yamagata lineage (31.8%) was observed during 2011–2012. From 2013 to February 2014, however, all isolates belonged to the B/Yamagata lineage. Although the 51 random isolates represented only one/tenth of all influenza B cases during this period, these data suggested a lineage shift from B/Victoria to B/Yamagata during the past 4 years in Thailand.

To fully characterize these circulating influenza B strains, the entire viral genomes of the 51 isolates were sequenced. For comparison, we also included 2 additional full-length influenza B sequences from samples previously isolated in 2006 and 2008. The designations, collection dates, and relevant details are shown in Table 2. Individual gene sequences from these 53 isolates were then compared to other strains previously isolated between 2010 to 2011 (GenBank accession numbers JX512971-JX513210), 2012 to 2014 (GenBank accession numbers KM100190-KM10033), and several established vaccine and reference strains.

**Table 2:** Influenza B virus clinical isolates sequenced in this study.

**Name of isolate**	**Collection date**	**Location**	**Age (yr)**	**Sex**	**Clade**	**Genes sequenced**
B/Thailand/CU-243/2006	2006	-	-	-	Vic-3	PB2, PB1, PA, HA, NP, NA, MP, NS
B/Thailand/CU-364/2008	2008	-	-	-	Vic-3	PB2, PB1, PA, HA, NP, NA, MP, NS
B/Thailand/CU-H1400/2010	1-Feb-10	Bangkok	6	Male	Vic-1B	PB2, PB1, PA, HA, NP, NA, MP, NS
B/Thailand/CU-B2201/2010	24-Feb-10	Bangkok	8	Male	Vic-1B	PB2, PB1, PA, HA, NP, NA, MP, NS
B/Thailand/CU-B2271/2010	5-Mar-10	Bangkok	5	Male	Vic-1A	PB2, PB1, PA, HA, NP, NA, MP, NS
B/Thailand/CU-B2320/2010	20-Mar-10	Bangkok	33	Male	Vic-1B	PB2, PB1, PA, HA, NP, NA, MP, NS
B/Thailand/CU-H1896/2010	29-Apr-10	Surat Thani	-	Female	Vic-1B	PB2, PB1, PA, HA, NP, NA, MP, NS
B/Thailand/CU-B2372/2010	4-Jun-10	Bangkok	17	Female	Vic-1B	PB2, PB1, PA, HA, NP, NA, MP, NS
B/Thailand/CU-B2390/2010	17-Jun-10	Bangkok	7	Female	Vic-5	PB2, PB1, PA, HA, NP, NA, MP, NS
B/Thailand/CU-B2432/2010	9-Jul-10	Bangkok	16	Female	Vic-1B	PB2, PB1, PA, HA, NP, NA, MP, NS
B/Thailand/CU-B2504/2010	9-Jul-10	Bangkok	6	Male	Vic-1A	PB2, PB1, PA, HA, NP, NA, MP, NS
B/Thailand/CU-H2132/2010	5-Aug-10	Surat Thani	-	Female	Vic-1B	PB2, PB1, PA, HA, NP, NA, MP, NS
B/Thailand/CU-B2660/2010	14-Aug-10	Bangkok	-	Female	Vic-1A	PB2, PB1, PA, HA, NP, NA, MP, NS
B/Thailand/CU-B3153/2010	1-Sep-10	Bangkok	14	Male	Vic-1A	PB2, PB1, PA, HA, NP, NA, MP, NS
B/Thailand/CU-H2584/2010	30-Sep-10	Bangkok	12	Female	Vic-1B	PB2, PB1, PA, HA, NP, NA, MP, NS
B/Thailand/CU-C1262/2010	5-Oct-10	Khon Kaen	10	Male	Vic-1A	PB2, PB1, PA, HA, NP, NA, MP, NS
B/Thailand/CU-H2738/2010	17-Nov-10	Bangkok	25	Male	Vic-1B	PB2, PB1, PA, HA, NP, NA, MP, NS
B/Thailand/CU-C1451/2010	8-Dec-10	Khon Kaen	14	Male	Vic-1A	PB2, PB1, PA, HA, NP, NA, MP, NS
B/Thailand/CU-B4504/2011	1-Jan-11	Bangkok	7	Female	Vic-1A	PB2, PB1, PA, HA, NP, NA, MP, NS
B/Thailand/CU-B4585/2011	4-Feb-11	Bangkok	33	Female	Vic-5	PB2, PB1, PA, HA, NP, NA, MP, NS
B/Thailand/CU-H2933/2011	12-Feb-11	Bangkok	3	Male	Yam-3	PB2, PB1, PA, HA, NP, NA, MP, NS
B/Thailand/CU-C1768/2011	22-Mar-11	Khon Kaen	-	Female	Vic-1A	PB2, PB1, PA, HA, NP, NA, MP, NS
B/Thailand/CU-H3002/2011	9-Jul-11	Bangkok	-	Male	Vic-1A	PB2, PB1, PA, HA, NP, NA, MP, NS
B/Thailand/CU-B5522/2011	27-Aug-11	Bangkok	7	Female	Vic-1A	PB2, PB1, PA, HA, NP, NA, MP, NS
B/Thailand/CU-H3052/2011	29-Aug-11	Bangkok	1	Male	Vic-1A	PB2, PB1, PA, HA, NP, NA, MP, NS
B/Thailand/CU-B5671/2011	15-Sep-11	Bangkok	34	Female	Vic-1B	PB2, PB1, PA, HA, NP, NA, MP, NS
B/Thailand/CU-B5734/2011	23-Sep-11	Bangkok	34	Female	Vic-1A	PB2, PB1, PA, HA, NP, NA, MP, NS
B/Thailand/CU-B5910/2011	4-Nov-11	Bangkok	50	Male	Vic-1B	PB2, PB1, PA, HA, NP, NA, MP, NS
B/Thailand/CU-B6078/2012	19-Feb-12	Bangkok	-	Male	Yam-2	PB2, PB1, PA, HA, NP, NA, MP, NS
B/Thailand/CU-B6096/2012	13-Mar-12	Bangkok	8	Male	Yam-3	PB2, PB1, PA, HA, NP, NA, MP, NS
B/Thailand/CU-B6148/2012	4-Apr-12	Bangkok	33	Male	Vic-1A	PB2, PB1, PA, HA, NP, NA, MP, NS
B/Thailand/CU-B6240/2012	23-May-12	Bangkok	30	Male	Vic-1B	PB2, PB1, PA, HA, NP, NA, MP, NS
B/Thailand/CU-B6257/2012	14-Jun-12	Bangkok	33	Female	Vic-1A	PB2, PB1, PA, HA, NP, NA, MP, NS
B/Thailand/CU-H3313/2012	16-Jul-12	Bangkok	9	Female	Yam-3	PB2, PB1, PA, HA, NP, NA, MP, NS
B/Thailand/CU-H3349/2012	20-Aug-12	Bangkok	2	Female	Yam-2	PB2, PB1, PA, HA, NP, NA, MP, NS
B/Thailand/CU-B6975/2012	20-Sep-12	Bangkok	8	Male	Vic-1A	PB2, PB1, PA, HA, NP, NA, MP, NS
B/Thailand/CU-H3456/2012	24-Oct-12	Bangkok	7	Female	Yam-2	PB2, PB1, PA, HA, NP, NA, MP, NS
B/Thailand/CU-B7215/2012	19-Nov-12	Bangkok	5	Male	Vic-1A	PB2, PB1, PA, HA, NP, NA, MP, NS
B/Thailand/CU-H3496/2012	14-Dec-12	Bangkok	11	Male	Yam-3	PB2, PB1, PA, HA, NP, NA, MP, NS
B/Thailand/CU-B7337/2012	18-Dec-12	Bangkok	55	Female	Vic-1A	PB2, PB1, PA, HA, NP, NA, MP, NS
B/Thailand/CU-B8332/2013	11-Feb-13	Bangkok	9	Female	Yam-2	PB2, PB1, PA, HA, NP, NA, MP, NS
B/Thailand/CU-B8813/2013	19-Dec-13	Bangkok	1	Male	Yam-2	PB2, PB1, PA, HA, NP, NA, MP, NS
B/Thailand/CU-A585/2013	25-Dec-13	Khon Kaen	56	Female	Yam-2	HA, NA, MP
B/Thailand/CU-A605/2014	7-Jan-14	Khon Kaen	39	Female	Yam-2	HA, NA, MP
B/Thailand/CU-B8925/2014	14-Jan-14	Bangkok	9	Male	Yam-2	PB2, PB1, PA, HA, NP, NA, MP, NS
B/Thailand/CU-H3591/2014	16-Jan-14	Bangkok	6	Female	Yam-2	PB2, PB1, PA, HA, NP, NA, MP, NS
B/Thailand/CU-A615/2014	22-Jan-14	Khon Kaen	55	Female	Yam-2	HA, NA
B/Thailand/CU-A626/2014	22-Jan-14	Khon Kaen	43	Female	Yam-2	HA, NA
B/Thailand/CU-B8999/2014	1-Feb-14	Bangkok	-	Female	Yam-2	PB2, PB1, PA, HA, NP, NA, MP, NS
B/Thailand/CU-B9017/2014	3-Feb-14	Bangkok	-	Female	Yam-2	HA, NA
B/Thailand/CU-A645/2014	5-Feb-14	Khon Kaen	54	Female	Yam-2	PB2, PB1, PA, HA, NP, NA, MP, NS
B/Thailand/CU-B9034/2014	7-Feb-14	Bangkok	40	Female	Yam-2	HA, NA
B/Thailand/CU-C4555/2014	19-Feb-14	Khon Kaen	4	Male	Yam-2	HA, NA, MP

### HA nucleotide sequence variations in clinical isolates

**Figure 3:**
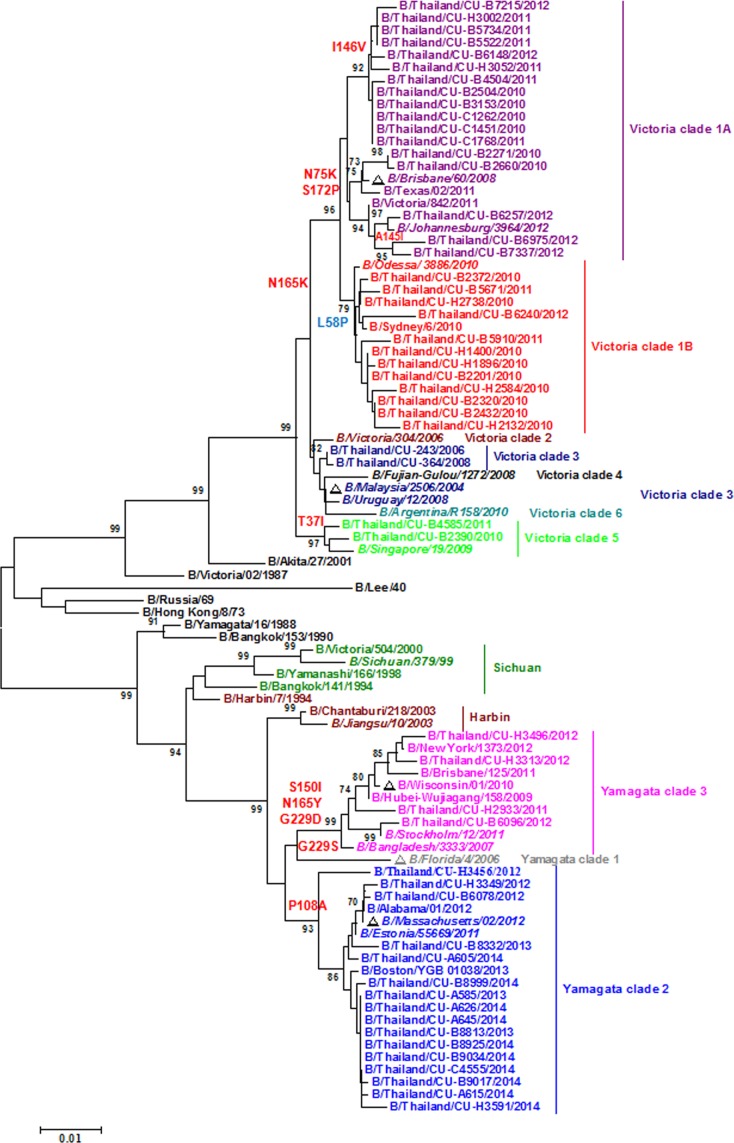
Phylogenetic analysis of the HA nucleotide sequences from influenza B strains isolated in Thailand from 2006–2014. The 53 HA sequences of influenza B (names beginning with B/Thailand/CU) were compared with those from the vaccine strains of southern hemisphere which are recommended by WHO (noted with triangles) and reference strains of the clades previously reported by WHO Influenza Centre London (italic font). The phylogenetic tree was generated by the neighbor-joining method with 1,000 bootstrap replicates. Branch values >70 are indicated. The scale bar represents approximately 1% nucleotide change between close relatives.

Phylogenetic analysis of the HA nucleotide sequences identified 6 genetic clades of influenza B/Victoria lineage and 3 genetic clades of the B/Yamagata lineage (Fig. 3). The majority of the clinical isolates grouped into B/Victoria clades 1A and 1B, especially isolates from 2010 to 2012. These clades shared the nucleotide sequences coding for the amino acid substitutions N165K, N75K, and S172P on the HA. Specifically, the B/Victoria clade 1A strains encoded an additional I146V substitution, while clade 1B strains encoded an additional L58P substitution. The B/Victoria clade 1A comprises the B/Brisbane/60/2008, a 2010–2012 Southern hemisphere vaccine strain. Moreover, 2 of the 51 isolates grouped into B/Victoria clade 5, which is characterized by the T37I substitution. Interestingly, the 2 isolates from 2006 and 2008 formed B/Victoria clade 3, as was B/Malaysia/2506/2004 included in the 2006 Southern hemisphere influenza vaccine (>99.3% nucleotide and amino acid similarities).

The remaining influenza B isolates clustered with B/Yamagata clades 2 and 3. Sixteen of the 51 isolates (99.31% nucleotides and 99.77% amino acid identity) clustered in clade 2, which is characterized by the P108A substitution. A notable strain in this clade included the B/Massachusetts/2/2012, which was chosen for the 2014 Southern hemisphere vaccine. Along with 4 isolates, clade 3 included the B/Wisconsin/01/2010 vaccine strain for 2013. Members in clade 3 possessed S150I, N165Y, and G229D substitutions and shared >99.2% nucleotide and amino acid homology.

### HA protein sequence variations in clinical isolates

As sequence variations on the HA protein can influence both receptor-binding and antigenic epitopes, we examined amino acid changes in these clinical isolates. Structural studies of the B/Hong Kong/8/73 HA protein, which represent an earlier strain before influenza B diverged into B/Victoria and B/Yamagata, had identified 4 major epitopes on HA1, the subunit of HA important for antigenic variation. They are the 120-loop, the 150-loop, the 160-loop, the 190-helix, and their respective surrounding regions [8]. The 33 HA sequences of B/Victoria lineage and 20 HA sequences of B/Yamagata lineage in this study showed diverse amino acid substitutions on these epitopes (Table 3). For example, the HA1 residues 179–181 near the 120-loop encode amino acids TKG (^179^TKG^181^) in strains isolated between 1972–1982 [24], but all isolates of the B/Victoria lineage in this study were ^179^TEG^181^. In addition, 4 of the B/Yamagata isolates also have ^179^TEG^181^, while 16 isolates have a novel ^179^AEG^181^. In the 150-loop, all B/Victoria strains have ^148^NGN^150^ similar to the early influenza B strains. However, 4 and 16 B/Yamagata isolates had ^148^SKI^150^ and ^148^SKS^150^, respectively. Within the 160-loop, isolates of both lineages had amino acid insertions in this region when compared to B/Hong Kong/8/73.

**Table 3:** Amino acid substitutions found in the HA protein of influenza B virus clinical isolates in this study^a^.

**Residues at site^b^**	**B/HK/73**	**Victoria (# of sequences)**	**Yamagata (# of sequences)**
**HA1 subunit**			
**120-Loop**			
48	Q	E (33)	R (4), K (16)
56	N	K (31), R (2)	D (20)
71	K	K (33)	M (20)
75	T	K (29), N (4)	T (19), I (1)
116	N	H (32), N (1)	K (2), N (18)
122	R	H (33)	Q (20)
125	T	I (33)	I (20)
129	T	N (32), S (1)	K (20)
179–181	TKG	TEG (33)	TEG (4), AEG (16)
**150-Loop**			
148–150	NGN	NGN (33)	SKI (4), SKS (16)
**160-Loop**			
Insertion at 162–163	-	NDK (30), NDN (3)	NDY (4), DNN (16)
**190-Helix**			
195	E	E (33)	K (20)
199	V	A (32), E (1)	K (20)
206	K	K (33)	N (20)
230	N	N (33)	D (20)
232	A	T (33)	T (9), R (11)
235	E	G (33)	G (20)
**Receptor binding site**			
136	I	K (33)	R (20)
**HA2 subunit**			
132	D	E (33)	D (20)
158	N	D (33)	N (20)

^a^ The defined residue positions on the antigenic epitope according to Ni et al., 2013 [24].

^b^ The residues are numbered according to that of B/HK/73 HA [23].

Comparison of the 33 isolates of B/Victoria lineage and 20 isolates of B/Yamagata lineage with the vaccine strains B/Brisbane/60/2008 (Victoria lineage) and B/Florida/4/2006 (Yamagata lineage) [26] showed identity at residues F95, W158, H191 and Y202 (S1 Fig.). These four amino acids form the base of the receptor-binding site on the HA protein and are highly conserved among all known sequences of influenza B virus HA [8]. However, all of our B/Yamagata isolates had I136R substitution, another receptor binding residue previously observed in 98% of B/Yamagata strains [10], while all isolates of B/Victoria lineage have I136K substitution. Finally, another important attribute of the HA protein is its glycosylation sequence. Our B/Yamagata isolates possessed a novel potential N-linked glycosylation at residue 197, which was not present in any of the vaccine or reference strains chosen for comparison.

### NA sequence variations in clinical isolates

**Figure 4:**
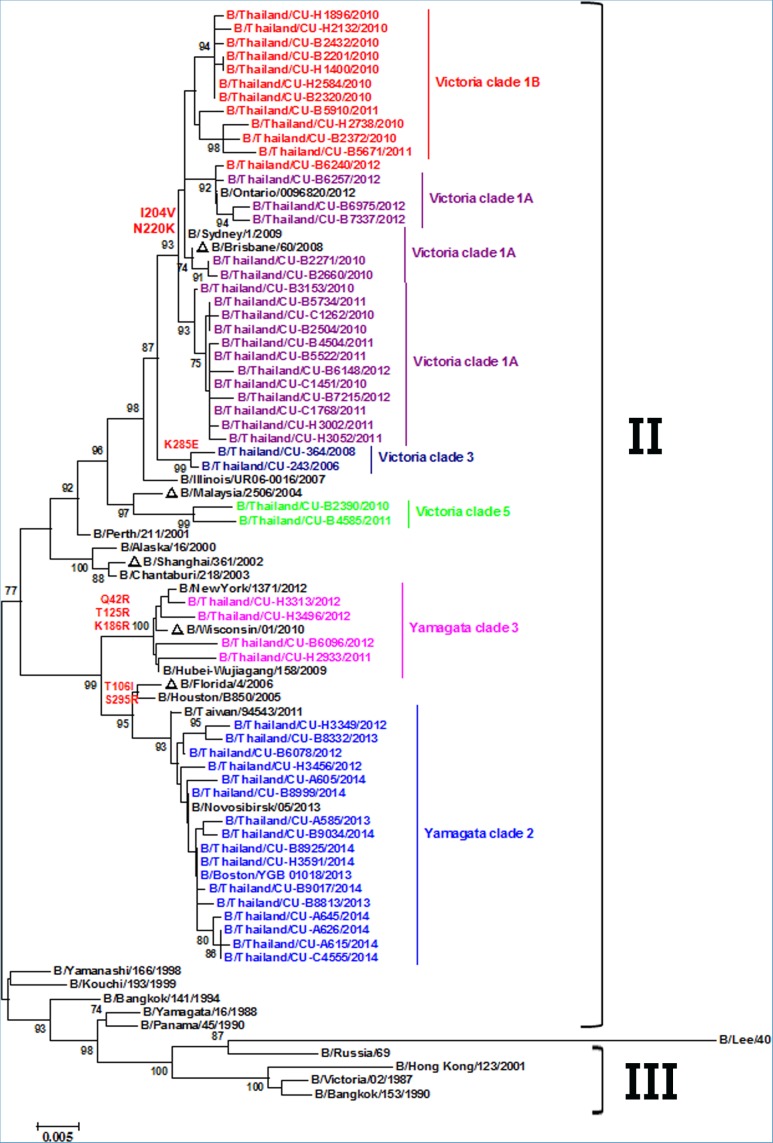
Phylogeny analysis of the NA nucleotide sequences from influenza B strains isolated in Thailand from 2010–2014. Trees were constructed using neighbor-joining method in MEGA (V.6.06). Bootstrap values (1,000 replicates) >70 are indicated on the branches. Analysis was based on nucleotide 1,402 base pairs. The scale bar represents approximately 0.5% nucleotide change between close relatives. The sequences isolated in this study are denoted by /Thailand/CU. The vaccine strains are preceded by open triangles.

Frequent reassortment of gene segments often complicates lineage assignment, therefore we analyzed the lineage in all subsequent gene segments using previously proposed group designation [12]. The phylogenetic tree of NA nucleotide sequences classified them into two distinct groups (groups II and III). Group II consisted of the viruses from HA Victoria and HA Yamagata lineages. All fifty-three isolates clustered in this group (Fig. 4). In contrast, group III consisted of viruses with the HA sequences belonging to Victoria lineage. The NA sequences of the B/Victoria clade 1 strains, which included the B/Brisbane/60/2008 vaccine, were characterized by I204V and N220K substitutions, whereas B/Victoria clade 3 strains contained K285E change. The remaining clinical isolates clustered within B/Yamagata clades 2 and 3. Clade 2, which included B/Florida/4/2006, was characterized by T106I and S295R substitutions, while clade 3, which included B/Wisconsin/1/2010, was distinguished by Q42R, T125K, and K186R substitutions. Therefore, comparative analysis of the NA sequences obtained from the clinical isolates differed from the HA phylogenetic tree.

There are several important conserved residues in the NA active site of influenza B virus [27]. The NA protein of influenza B clinical isolates have eight catalytic residues (R116, D149, R150, R223, E275, R292, R374, and Y409) and 11 framework residues (E117, R154, W177, S178, D197, I221, E226, H273, E276, N293, E428) (S1 Fig.). None of the 53 NA protein sequences we analyzed displayed substitutions in the active site and their surrounding residues. In addition to the 4 recognized potential N-linked glycosylation sites on the NA protein at positions 56, 64, 144, and 284, 5 of our isolates have a new glycosylation site at residue 463.

### Other influenza B gene segments

For the three influenza B polymerase genes (PB1, PB2, and PA), we were able to obtain full sequences from 46 strains. The polymerase genes are classified into three distinct groups (I, II, and III). Based on PB1 and PB2, all Yamagata and Victoria strains belonged to groups II and III, respectively. Phylogenetic trees of the PB2 and PB1 gene sequences appeared very similar (S2 and S3 Figs., respectively). The PA and the NP genes of either Victoria and Yamagata strains, however, all clustered in group II (S4 and S5 Figs.). For the M gene, we were able to obtain sequences from 49 strains. Its phylogenetic tree also showed that all isolates clustered into group II (S6 Fig.). Since both Victoria and Yamagata isolates clustered into the same group II based on the phylogenetic analysis of the PA, NP, and M genes, it appeared likely that even after gene reassortment by some clinical isolates these genes remained associated. Furthermore, analysis of the 46 NS gene sequences in this study indicated that while the reference strains B/Victoria/02/1987 and B/Yamagata/16/1988 formed group III, all of the clinical isolates clustered together to form a separate group IV (S7 Fig.).

A summary of the genetic analysis for each gene segment is shown in Table 4. The six internal genes of influenza B virus displayed three distinct evolutionary profiles: (i) PB1 and PB2 genes; (ii) PA, NP and M genes; and (iii) NS gene. This observation suggests that the PA, NP, M, and NS genes evolved and reassorted independently of the HA gene.

**Table 4:** Summary of the whole genome analysis and phylogenetic patterns of influenza B virus isolated in Thailand between 2010–2014.

**No. of strain (Year of isolation)**	**HA**	**NA**	**PB1**	**PB2**	**PA**	**NP**	**M**	**NS**
2 (2006–2008)	Vic-3	II	III	III	II	II	II	IV
2 (2010–2011)	Vic-5	II	III	III	II	II	II	IV
17 (2010–2012)	Vic-1A	II	III	III	II	II	II	IV
12 (2010–2012)	Vic-1B	II	III	III	II	II	II	IV
4 (2011–2012)	Yam-3	II	II	II	II	II	II	IV
9 (2012–2014)	Yam-2	II	II	II	II	II	II	IV

Vic, strains clustered with B/Victoria/2/87; Yam, strains grouped in B/Yamagata/16/88.

### Overall profile of amino acid variations observed among the clinical isolates

**Figure 5:**
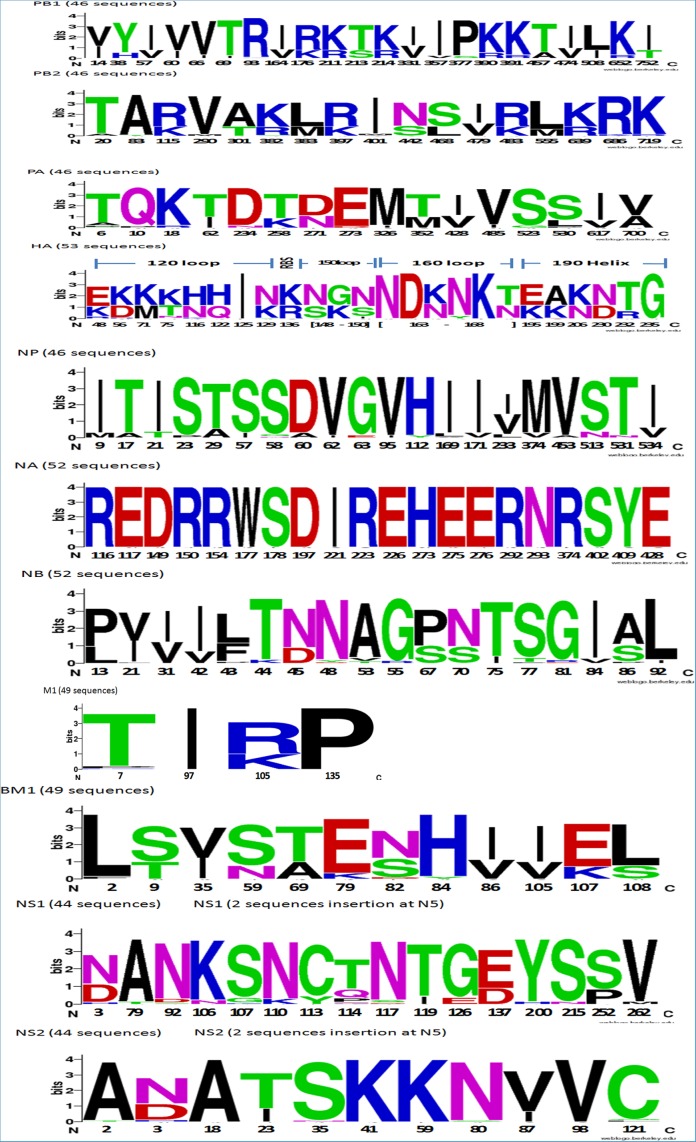
Amino acid residues of influenza B virus proteins isolated in Thailand during 2010–2014. (A) PB1, (B) PB2, (C) PA, (D) HA, (E) NP, (F) NA, (G) NB, (H) M1, (I) BM1, (J) NS1 and (K) NS2. The graphics were generated using WebLogo3. The relative frequency of the corresponding amino acid at a given position is proportional to the residue height. Residue positions are indicated on the x-axis. Amino acids are colored according to their chemical properties: polar amino acids (G,S,T,Y,C) are green, basic (K,R,H) blue, acidic (D,E) red, amide polar (Q, N) purple, and hydrophobic (A,V,L,I,P,W,F,M) amino acids are black.

A number of previous studies have described and characterized point mutations in the genome of influenza B viruses (6, 24, 26, 44–45). To yield a better understanding of the total amino acid mutations found in all viral genes among our isolates, all 378 sequenced reads obtained from all genes were analyzed. The amino acid substitutions in the PB1, PB2, PA, HA, NP, NA, NB, M1, BM1, NS1, and NS2 are summarized (Fig. 5). PB1, PB2, PA, NB, and BM1 displayed intermediate variation throughout their protein sequences, while the HA protein accumulated the most diversity of amino acid changes. In contrast, the NA protein appeared the most constant among the influenza B proteins.

## Discussions

In this study, we surveyed the incidence of influenza B virus from 14,418 respiratory tract samples obtained between 2010 to 2014. We found an annual rate of influenza B infection in Thailand averaged 3.27% during this period similar to the rate of 3.68% found in the U.S. [28] and 3.0% in Singapore [29]. The majority of influenza B virus infection affected children and adolescent between 5–19 years, which was reflective of the epidemiological data in the Japan [30] and Finland [31].

Although influenza infection can occur year-round, environmental factors can influence host susceptibility and increase viral spread [32–34]. The current study suggested that the relative humidity and rainfall was positively correlated with a higher prevalence of influenza B cases [data not shown] as was seen in an earlier study in Thailand [20] and Hong Kong [35]. Rain and cold weather have also been associated with seasonal influenza transmission due to close-contact and contribute to an increased risk of person-to-person transmission [36]. The seasonal patterns observed in this study further support a proposed influenza vaccination schedule of March and April in Thailand [20].

Our whole genome sequences contribute to the growing collection of the most recent circulating strains of influenza B in Southeast Asia. Although randomly sampling one clinical isolate per month may be too few, the available data revealed changing patterns of prevalence for influenza A and B viruses in Thailand. We observed that the types or subtypes of influenza A (H1N1 pdm09, or H3N2) and B viruses have fluctuated over the past four seasons. When an influenza A virus season is severe and prolonged, influenza B diversity and severity were generally reduced [12]. This might have been the case in 2013 when we observed an extremely low rate of influenza B (0.37%) despite the high A/H3N2 infection in the same period. In addition, A/H1N1 pdm09 virus emerged worldwide since 2009 and this infection peaked in Thailand dramatically in 2010 until mid-2011. This might have contributed to the low level of influenza B cases in 2011.

The molecular characterization of eight gene segments of influenza B virus was previously classified [9,37,38], but the tendency for influenza to reassort complicates viral classification. Therefore, genes other than HA were classified into groups. Five internal genes and NA could be broadly classified into three groups (I, II, and III), while the NS gene could be characterized into four groups (I–IV). Phylogenetic division of HA (6 Victoria and 3 Yamagata clades) reveals that the circulating influenza B strains during this time were Victoria clades 1A, 1B, 3, 5 and Yamagata clades 2 and 3. All of the NA sequences belonged to group II, the same group as B/Yamagata/88. This suggests that reassortment of the HA and NA genes may have occurred consistent with a previous report [38]. Meanwhile, the phylogeny of PB1 and PB2 genes clustered into group II and III, which correspond with the HA of B/Yamagata/88 and B/Victoria/87 lineages, respectively. The evolution of the PB1 and PB2 genes were similar to the HA gene [38]. In contrast, all of the PA, NP, and M genes, regardless of the lineage, were in group II. This suggests reassortment of these genes in influenza B virus [11]. The PA, NP, and M genes had similar evolutionary patterns suggesting possible functional association among the proteins [15]. The NS gene sequences belonging to both HA Victoria and Yamagata lineages were classified into group IV, and phylogeny of the NS tree was absolutely divergent compared to the NP and M genes. Therefore, the NS gene exhibited the pattern of genetic reassortment which was distinct from those of the NP or M genes.

The N-linked glycosylation plays a major role in stabilizing the HA structure, to protect the HA protein from being hydrolyzed by the enzyme and to evade antibody recognition. The diversity in glycosylation on the HA1 epitope is known to result in an antigenic change [39]. In this study, the N-linked glycosylation at residue N123 on HA1 no longer existed in either the B/Yamagata and B/Victoria lineage strains due to T125I substitution. This residue is located on the strong antigenic determinant 120-loop and the alteration may help the virus escape neutralizing antibody from the host [10]. Meanwhile, the B/Victoria lineage strains have a glycosylation site at HA1 233 shared by none of the B/Yamagata lineage strains.

The four major antigenic sites of influenza B HA are on the 120-loop, the 150-loop, the 160-loop and the 190-helix and their surrounding regions [39]. Analysis of the HA sequences showed frequent amino acid substitution on these four major epitope residues. The 120-loop epitope dictates the antigenicity of HA1 and as a result most of the mutations were found here. Fewer substitutions were observed on the 150-loop and the 160-loop among the isolates. The numbers of amino acid mutation contributing to the HA structure were sufficiently large enough to escape antigen recognition of neutralizing antibodies, but still small enough to maintain the structural integrity of the protein, especially the receptor-binding site to ensure potential binding of host cell receptors [10].

The NA active site contains 19 highly conserved residues common to all influenza A and B viruses [27]. A total of 53 NA protein sequences have eight catalytic residues (R116, D149, R150, R223, E275, R292, R374, and Y409) that directly contact the sialic acid and 11 framework residues (E117, R154, W177, S178, D197, I221, E226, H273, E276, N293, E428) that support the enzymatic binding pocket. Reports indicated that E117V/A, D197N/E/Y, I221T, H273Y, R292K, and R374K mutations of influenza B NA could contribute to reduced susceptibility of oseltamivir and zanamivir [40–42]. The NA protein sequences obtained from all of the influenza B isolates did not have any changes in the active site or their surrounding residues, therefore these strains likely remained susceptible to neuraminidase inhibitor.

The existing influenza B vaccine has the limitation in that it does not cross-protect between the two distinct influenza B lineages and as a result the vaccine efficacy decreased when the included vaccine strain did not match the circulating epidemic strain [43]. The influenza vaccine recommended by WHO and used in Thailand from 2010 to 2012 comprised of only the B/Victoria/87 lineage (B/Brisbane/60/2008), and although the sequence closely matched that of our clinical isolates, it did not protect infection by the B/Yamagata strains. This may explain why we observed an increased incidence of B/Yamagata (8.3%) in 2012. Thus, a quadri-valent influenza vaccine that consists of H1N1, H3N2 and two lineages of influenza B viruses should be recommended to better provide protection from influenza B infection. In summary, the phylogenetic tree of all gene segments and the mutations identified at various positions on epitopes will provide a better understanding of influenza B evolution and lead to a better vaccine development strategy.

## Supporting Information

S1 Fig Haemagglutinin and neuraminidase protein sequence alignments for influenza B virus isolates. (PDF)

S2 Fig Phylogenetic trees of PB2 gene of influenza B viruses of 44 samples isolated in Thailand, 2010–2014. Trees were constructed using Neighbor Joining analysis in MEGA (V.6.06). Bootstrap values (1000 replicates) > 70 are indicated on the branch. The scale bar shows the mutation rate between each two sequences. The sequences studied in this study are represented by name of taxa “Thailand”. All vaccine strains are marked as open triangles.
(TIF)

S3 Fig Phylogenetic trees of PB1 gene of influenza B viruses of 44 samples isolated in Thailand, 2010–2014. Trees were constructed using Neighbor Joining analysis in MEGA (V.6.06). Bootstrap values (1000 replicates) > 70 are indicated on the branch. The scale bar shows the mutation rate between each two sequences. The sequences studied in this study are represented by name of taxa “Thailand”. All vaccine strains are marked as open triangles.
(TIF)

S4 Fig Phylogenetic trees of PA gene of influenza B viruses of 44 samples isolated in Thailand, 2010–2014. Trees were constructed using Neighbor Joining analysis in MEGA (V.6.06). Bootstrap values (1000 replicates) > 70 are indicated on the branch. The scale bar shows the mutation rate between each two sequences. The sequences studied in this study are represented by name of taxa “Thailand”. All vaccine strains are marked as open triangles.
(TIF)

S5 Fig Phylogenetic trees of NP gene of influenza B viruses of 44 samples isolated in Thailand, 2010–2014. Trees were constructed using Neighbor Joining analysis in MEGA (V.6.06). Bootstrap values (1000 replicates) > 70 are indicated on the branch. The scale bar shows the mutation rate between each two sequences. The sequences studied in this study are represented by name of taxa “Thailand”. All vaccine strains are marked as open triangles.
(TIF)

S6 Fig Phylogenetic trees of M gene of influenza B viruses of 47 samples isolated in Thailand, 2010–2014. Trees were constructed using Neighbor Joining analysis in MEGA (V.6.06). Bootstrap values (1000 replicates) > 70 are indicated on the branch. The scale bar shows the mutation rate between each two sequences. The sequences studied in this study are represented by name of taxa “Thailand”. All vaccine strains are marked as open triangles.
(TIF)

S7 Fig Phylogenetic trees of NS gene of influenza B viruses of 44 samples isolated from Thailand, 2010–2014. Trees were constructed using Neighbor Joining analysis in MEGA (V.6.06). Bootstrap values (1000 replicates) > 70 are indicated on the branch. The scale bar shows the mutation rate between each two sequences. The sequences studied in this study are represented by name of taxa “Thailand”. All vaccine strains are marked as open triangles.
(TIF)

S1 Table Primer sets used for conventional PCR amplification of the whole genome of influenza B isolates. (DOCX)

S2 Table Accession numbers of influenza B sequences in GenBank and GISAID used to construct phylogenetic trees of 8 genes. (DOCX)
