# Supplementary material for: Molecular Epidemiology and Phylogenetic Analyses of Influenza B Virus in Thailand during 2010 to 2014
Source: PLoS One. 2015 Jan 20;10(1):e0116302. doi: 10.1371/journal.pone.0116302 (PMC4300180; doi:10.1371/journal.pone.0116302)
Supplement: S1 Fig — (PDF) [file pone.0116302.s001.pdf]

# HA protein alignment for B/Victoria lineage strains in Thailand, 2010 – 2014

Reference strains, Vaccine strains, Thailand strains; Potential N-linked glycosylation sites; Receptor binding site

```
                                 HA1     10        20        30        40        50        60        70        80        90       100
                                 ....|....|....|....|....|....|....|....|....|....|....|....|....|....|....|....|....|....|....|....|
B/Malaysia/2506/2004             DRICTGITSSNSPHVVKTATQGEVNVTGVIPLTTTPTKSHFANLKGTETRGKLCPKCLNCTDLDVALGRPKCTGNIPSARVSILHEVRPVTSGCFPIMHD
B/Thailand/CU-243/2006           ....................................................................................................
B/Thailand/CU-364/2008           ....................................................................................................
B/Singapore/19/2009              ....................................I...............................................................
B/Thailand/CU-B2390/2010         ....................................I...............................................................
B/Thailand/CU-B4585/2011         ....................................I...............................................................
B/Brisbane/60/2008               ..........................................................................K.........................
B/Johannesburg/3964/2012         ..............................................A...........................K.........................
B/Thailand/CU-B2271/2010         .......................................................R..................K.........................
B/Thailand/CU-B2660/2010         .......................................................R..................K.........................
B/Thailand/CU-B2504/2010         ..........................................................................K.........................
B/Thailand/CU-B3153/2010         ..........................................................................K.........................
B/Thailand/CU-B4504/2011         ..........................................................................K.........................
B/Thailand/CU-B5522/2011         ..........................................................................K.........................
B/Thailand/CU-C1262/2010         ..........................................................................K.........................
B/Thailand/CU-C1451/2010         ..........................................................................K.........................
B/Thailand/CU-C1768/2011         ..........................................................................K.........................
B/Thailand/CU-H3002/2011         ..........................................................................K.........................
B/Thailand/CU-H3052/2011         ..........................................................................K.........................
B/Thailand/CU-B6148/2012         ..........................................................................K.........................
B/Thailand/CU-B6257/2012         ..........................................................................K.........................
B/Thailand/CU-B6975/2012         ..........................................................................K.........................
B/Thailand/CU-B7215/2012         ..........................................................................K.........................
B/Thailand/CU-B7337/2012         ..........................................................................K.........................
B/Thailand/CU-B5734/2011         ..........................................................................K.........................
B/Odessa/3886/2010               .........................................................P................K.........................
B/Thailand/CU-B2201/2010         .........................................................P................K.........................
B/Thailand/CU-B2320/2010         .........................................................P................K.........................
B/Thailand/CU-B2372/2010         .........................................................P................K.........................
B/Thailand/CU-B2432/2010         .........................................................P................K.........................
B/Thailand/CU-B5671/2011         .........................................................P................K.........................
B/Thailand/CU-H2738/2010         .........................................................P................K.........................
B/Thailand/CU-B5910/2011         .........................................................P................K.........................
B/Thailand/CU-B6240/2012         .........................................................P................K...........I.............
B/Thailand/CU-H1400/2010         .........................................................P................K.........................
B/Thailand/CU-H1896/2010         .........................................................P..............M.K.........................
B/Thailand/CU-H2132/2010         .........................................................P................K.........................
B/Thailand/CU-H2584/2010         .........................................................P................K.....................M...
```

```
                                        110       120       130       140       150       160       170       180       190       200
                                 ....|....|....|....|....|....|....|....|....|....|....|....|....|....|....|....|....|....|....|....|
B/Malaysia/2506/2004             RTKIRQLPNLLRGYEHIRLSTHNVINAENAPGGSYKIGTSGSCPNVTNGNGFFATMAWAVPKNDNNKTATNSLTIEVPYICTEGEDQITVWGFHSDNEAQ
B/Thailand/CU-243/2006           ..................................................................................................T.
B/Thailand/CU-364/2008           ..................................................................................................T.
B/Singapore/19/2009              ..................................................................................................T.
B/Thailand/CU-B2390/2010         ...................................................................A..............................T.
B/Thailand/CU-B4585/2011         ................................................................K......P..........................T.
B/Brisbane/60/2008               .............................................I..................K......P........................D.T.
B/Johannesburg/3964/2012         ................................................................K......P..........................T.
B/Thailand/CU-B2271/2010         .............................................I..................K......P..........................T.
B/Thailand/CU-B2660/2010         .............................................I..................K......P..........................T.
B/Thailand/CU-B2504/2010         ................................................................K......P..........................T.
B/Thailand/CU-B3153/2010         ................................................................K......P..........................T.
B/Thailand/CU-B4504/2011         ................................................................K......P..........................T.
B/Thailand/CU-B5522/2011         .....................................................E..........K......P..........................T.
B/Thailand/CU-C1262/2010         ................................................................K......P..........................T.
B/Thailand/CU-C1451/2010         ................................................................K......P..........................T.
B/Thailand/CU-C1768/2011         ................................................................K......P..........................T.
B/Thailand/CU-H3002/2011         .....................................................E..........K......P.................I........T.
B/Thailand/CU-H3052/2011         .................K...................................E..........K......P..........................T.
B/Thailand/CU-B6148/2012         .....................................................E..........K......P..........................T.
B/Thailand/CU-B6257/2012         ................................................................K......P..........................T.
B/Thailand/CU-B6975/2012         .....................................................T..........K......P..........................T.
B/Thailand/CU-B7215/2012         .....................................................E..........K......P..........................T.
B/Thailand/CU-B7337/2012         .....................................................T..........K......P..........................T.
B/Thailand/CU-B5734/2011         .....................................................E..........K......P..........................T.
B/Odessa/3886/2010               ................................................................K......P..........................T.
B/Thailand/CU-B2201/2010         ................................................................K......P..........................T.
B/Thailand/CU-B2320/2010         ................................................................K......P..........................T.
B/Thailand/CU-B2372/2010         ................................................................K......P..........................T.
B/Thailand/CU-B2432/2010         ................................................................K......P..........................T.
B/Thailand/CU-B5671/2011         ................................................................K......P..........................T.
B/Thailand/CU-H2738/2010         ................................................................K......P..........................T.
B/Thailand/CU-B5910/2011         ................................................................K......P..........................T.
B/Thailand/CU-B6240/2012         ...............N............S...................................K.....DP..........................T.
B/Thailand/CU-H1400/2010         ................................................................K......P..........................T.
B/Thailand/CU-H1896/2010         ................................................................K......P..........................T.
B/Thailand/CU-H2132/2010         ................................................................K......P..........................T.
B/Thailand/CU-H2584/2010         .....................................................E..........K......P..........................T.
```

```
                                210       220       230       240       250       260       270       280       290       300
                        ....|....|....|....|....|....|....|....|....|....|....|....|....|....|....|....|....|....|....|....|
B/Malaysia/2506/2004    MAKLYGDSKPQKFTSSANGVTTHYVSQIGGFPNQTEDGGLPQSGRIVVDYMVQKSGKTGTITYQRGILLPQKVWCASGRSKVIKGSLPLIGEADCLHEKY
B/Thailand/CU-243/2006  ....................................................................................................
B/Thailand/CU-364/2008  ....................................................................................................
B/Singapore/19/2009     ....................................................................................................
B/Thailand/CU-B2390/2010 ....................................................................................................
B/Thailand/CU-B4585/2011 .............................................................................N......................
B/Brisbane/60/2008      ....................................................................................................
B/Johannesburg/3964/2012 ....................................................................................................
B/Thailand/CU-B2271/2010 ....................................................................................................
B/Thailand/CU-B2660/2010 ......................................................Y.............................................
B/Thailand/CU-B2504/2010 ....................................................................................................
B/Thailand/CU-B3153/2010 ....................................................................................................
B/Thailand/CU-B4504/2011 ....................................................................................................
B/Thailand/CU-B5522/2011 ....................................................................................................
B/Thailand/CU-C1262/2010 ....................................................................................................
B/Thailand/CU-C1451/2010 ....................................................................................................
B/Thailand/CU-C1768/2011 ....................................................................................................
B/Thailand/CU-H3002/2011 ....................................................................................................
B/Thailand/CU-H3052/2011 ....................................................................................................
B/Thailand/CU-B6148/2012 .......................................................E............................................
B/Thailand/CU-B6257/2012 ....................................................................................................
B/Thailand/CU-B6975/2012 ....................................................................................................
B/Thailand/CU-B7215/2012 ....................................................................................................
B/Thailand/CU-B7337/2012 ....................................................................................................
B/Thailand/CU-B5734/2011 ....................................................................................................
B/Odessa/3886/2010      ....................................................................................................
B/Thailand/CU-B2201/2010 ....................................................................................................
B/Thailand/CU-B2320/2010 ....................................................................................................
B/Thailand/CU-B2372/2010 ...................................................M................................................
B/Thailand/CU-B2432/2010 ....................................................................................................
B/Thailand/CU-B5671/2011 .E..................................................................................................
B/Thailand/CU-H2738/2010 ....................................................................................................
B/Thailand/CU-B5910/2011 ....................................................................................................
B/Thailand/CU-B6240/2012 ....................................................................................................
B/Thailand/CU-H1400/2010 ....................................................................................................
B/Thailand/CU-H1896/2010 ....................................................................................................
B/Thailand/CU-H2132/2010 ........................................S...........................................................
B/Thailand/CU-H2584/2010 ....................................................................................................
```

```
                                310       320       330       340       HA2       360       370       380       390       400
                        ....|....|....|....|....|....|....|....|....|....|....|....|....|....|....|....|....|....|....|....|
B/Malaysia/2506/2004    GGLNKSKPYYTGEHAKAIGNCPIWVKTPLKLANGTKYRPPAKLLKERGFFGAIAGFLEGGWEGMIAGWHGYTSHGAHGVAVAADLKSTQEAINKITKNLN
B/Thailand/CU-243/2006  ....................................................................................................
B/Thailand/CU-364/2008  ....................................................................................................
B/Singapore/19/2009     ....................................................................................................
B/Thailand/CU-B2390/2010 ....................................................................................................
B/Thailand/CU-B4585/2011 ....................................................................................................
B/Brisbane/60/2008      ....................................................................................................
B/Johannesburg/3964/2012 ....................................................................................................
B/Thailand/CU-B2271/2010 ....................................................................................................
B/Thailand/CU-B2660/2010 ....................................................................................................
B/Thailand/CU-B2504/2010 ....................................................................................................
B/Thailand/CU-B3153/2010 ....................................................................................................
B/Thailand/CU-B4504/2011 ....................................................................................................
B/Thailand/CU-B5522/2011 ....................................................................................................
B/Thailand/CU-C1262/2010 ....................................................................................................
B/Thailand/CU-C1451/2010 ....................................................................................................
B/Thailand/CU-C1768/2011 ....................................................................................................
B/Thailand/CU-H3002/2011 ....................................................................................................
B/Thailand/CU-H3052/2011 ....................................................................................................
B/Thailand/CU-B6148/2012 ....................................................................................................
B/Thailand/CU-B6257/2012 ....................................................................................................
B/Thailand/CU-B6975/2012 ....................................................................................................
B/Thailand/CU-B7215/2012 ....................................................................................................
B/Thailand/CU-B7337/2012 ....................................................................................................
B/Thailand/CU-B5734/2011 ....................................................................................................
B/Odessa/3886/2010      ....................................................................................................
B/Thailand/CU-B2201/2010 ....................................................................................................
B/Thailand/CU-B2320/2010 ....................................................................................................
B/Thailand/CU-B2372/2010 ....................................................................................................
B/Thailand/CU-B2432/2010 ....................................................................................................
B/Thailand/CU-B5671/2011 ....................................................................................................
B/Thailand/CU-H2738/2010 ....................................................................................................
B/Thailand/CU-B5910/2011 ....................................................................................................
B/Thailand/CU-B6240/2012 ....................................................................................................
B/Thailand/CU-H1400/2010 ....................................................................................................
B/Thailand/CU-H1896/2010 ....................................................................................................
B/Thailand/CU-H2132/2010 ....................................................................................................
B/Thailand/CU-H2584/2010 ....................................................................................................
```

```
                                 410       420       430       440       450       460       470       480       490       500
                          ....|....|....|....|....|....|....|....|....|....|....|....|....|....|....|....|....|....|....|....|
B/Malaysia/2506/2004      SLSELEVKNLQRLSGAMDELHNEILELDEKVDDLRADTISSQIELAVLLSNEGIINSEDEHLLALERKLKKMLGPSAVEIGNGCFETKHKCNQTCLDRIA
B/Thailand/CU-243/2006    ....................................................................................................
B/Thailand/CU-364/2008    ....................................................................................................
B/Singapore/19/2009       ....................................................................................................
B/Thailand/CU-B2390/2010  ....................................................................................................
B/Thailand/CU-B4585/2011  ....................................................................................................
B/Brisbane/60/2008        ....................................................................................................
B/Johannesburg/3964/2012  ....................................................................................................
B/Thailand/CU-B2271/2010  ....................................................................................................
B/Thailand/CU-B2660/2010  ....................................................................................................
B/Thailand/CU-B2504/2010  ....................................................................................................
B/Thailand/CU-B3153/2010  ....................................................................................................
B/Thailand/CU-B4504/2011  ....................................................................................................
B/Thailand/CU-B5522/2011  ....................................................................................................
B/Thailand/CU-C1262/2010  ....................................................................................................
B/Thailand/CU-C1451/2010  .............................................................................I......................
B/Thailand/CU-C1768/2011  ....................................................................................................
B/Thailand/CU-H3002/2011  ....................................................................................................
B/Thailand/CU-H3052/2011  ....................................................................................................
B/Thailand/CU-B6148/2012  ....................................................................................................
B/Thailand/CU-B6257/2012  ....................................................................................................
B/Thailand/CU-B6975/2012  ....................................................................................................
B/Thailand/CU-B7215/2012  ....................................................................................................
B/Thailand/CU-B7337/2012  ....................................................................................................
B/Thailand/CU-B5734/2011  ....................................................................................................
B/Odessa/3886/2010        ....................................................................................................
B/Thailand/CU-B2201/2010  ....................................................................................................
B/Thailand/CU-B2320/2010  ....................................................................................................
B/Thailand/CU-B2372/2010  ....................................................................................................
B/Thailand/CU-B2432/2010  ....................................................................................................
B/Thailand/CU-B5671/2011  ....................................................................................................
B/Thailand/CU-H2738/2010  ....................................................................................................
B/Thailand/CU-B5910/2011  ....................................................................................................
B/Thailand/CU-B6240/2012  ....................................................................................................
B/Thailand/CU-H1400/2010  ....................................................................................................
B/Thailand/CU-H1896/2010  ....................................................................................................
B/Thailand/CU-H2132/2010  ....................................................................................................
B/Thailand/CU-H2584/2010  ....................................................................................................
```

```
                                 510       520       530       540       550       560       570
                          ....|....|....|....|....|....|....|....|....|....|....|....|....|....|.
B/Malaysia/2506/2004      AGTFDAGEFSLPTFDSLNITAASLNDDGLDNHTILLYYSTAASSLAVTLMIAIFVVYMVSRDNVSCSICL*
B/Thailand/CU-243/2006    ......................................................................*
B/Thailand/CU-364/2008    ......................................................................*
B/Singapore/19/2009       ......................................................................*
B/Thailand/CU-B2390/2010  ......................................................................*
B/Thailand/CU-B4585/2011  ......................................................................*
B/Brisbane/60/2008        ......................................................................*
B/Johannesburg/3964/2012  ......................................................................*
B/Thailand/CU-B2271/2010  ......................................................................*
B/Thailand/CU-B2660/2010  ......................................................................*
B/Thailand/CU-B2504/2010  ..........................................................I...........*
B/Thailand/CU-B3153/2010  ..........................................................I...........*
B/Thailand/CU-B4504/2011  ..........................................................I...........*
B/Thailand/CU-B5522/2011  ..........................................................I...........*
B/Thailand/CU-C1262/2010  ..........................................................I...........*
B/Thailand/CU-C1451/2010  ..........................................................I...........*
B/Thailand/CU-C1768/2011  ..........................................................I...........*
B/Thailand/CU-H3002/2011  ..........................................................I...........*
B/Thailand/CU-H3052/2011  ..........................................................I...........*
B/Thailand/CU-B6148/2012  ..........................................................I...........*
B/Thailand/CU-B6257/2012  ......................................................................*
B/Thailand/CU-B6975/2012  ......................................................................*
B/Thailand/CU-B7215/2012  ..........................................................I...........*
B/Thailand/CU-B7337/2012  ......................................................................*
B/Thailand/CU-B5734/2011  ..........................................................I...........*
B/Odessa/3886/2010        ......................................................................*
B/Thailand/CU-B2201/2010  ......................................................................*
B/Thailand/CU-B2320/2010  ......................................................................*
B/Thailand/CU-B2372/2010  ......................................................................*
B/Thailand/CU-B2432/2010  ......................................................................*
B/Thailand/CU-B5671/2011  ......................................................................*
B/Thailand/CU-H2738/2010  ......................................................................*
B/Thailand/CU-B5910/2011  ......................................................................*
B/Thailand/CU-B6240/2012  ......................P...............................................*
B/Thailand/CU-H1400/2010  ......................................................................*
B/Thailand/CU-H1896/2010  ......................................................................*
B/Thailand/CU-H2132/2010  ......................................................................*
B/Thailand/CU-H2584/2010  ......................................................................*
```

# HA protein alignment for B/Yamagata lineage strains in Thailand, 2010 – 2014

Reference strains, Vaccine strains, Thailand strains; Potential N-linked glycosylation sites; Receptor binding site

```
                          HA1      10        20        30        40        50        60        70        80        90       100
                          ....|....|....|....|....|....|....|....|....|....|....|....|....|....|....|....|....|....|....|....|
B/Florida/4/2006          DRICTGITSSNSPHVVKTATQGEVNVTGVIPLTTTPTKSYFANLKGTRTRGKLCPDCLNCTDLDVALGRPMCVGTTPSAKASILHEVKPVTSGCFPIMHD
B/Wisconsin/1/2010        .......................................................................................R............
B/Bangladesh/3333/2007    .......................................................................................R............
B/Stockholm/12/2011       ............................A..........................................................R............
B/Thailand/CU-H2933/2011  ......................................................................................IR............
B/Thailand/CU-B3313/2012  .......................................................................................R............
B/Thailand/CU-H3496/2012  .......................................................................................R............
B/Thailand/CU-B6096/2012  ............................A..........................................................R............
B/Massachusetts/2/2012    ...............................................K.......................................R............
B/Estonia/55669/2011      ...............................................K.......................................R............
B/Thailand/CU-H3349/2012  ...............................................K.......................................R............
B/Thailand/CU-H3456/2012  ...............................................K.....................I.................R............
B/Thailand/CU-B6078/2011  ...............................................K.......................................R............
B/Thailand/CU-A585/2013   ...............................................K.......................................R............
B/Thailand/CU-B8813/2013  ...............................................K.......................................R............
B/Thailand/CU-B8332/2013  ...............................................K.......................................R............
B/Thailand/CU-A605/2014   ...............................................K.......................................R............
B/Thailand/CU-A615/2014   ..............M................................K.......................................R............
B/Thailand/CU-A626/2014   ...............................................K.......................................R............
B/Thailand/CU-A645/2014   ...............................................K.......................................R............
B/Thailand/CU-B8925/2014  ...............................................K.......................................R............
B/Thailand/CU-B8999/2014  ...............................................K.......................................R............
B/Thailand/CU-B9017/2014  ...............................................K.......................................R............
B/Thailand/CU-B9034/2014  ...............................................K.......................................R............
B/Thailand/CU-C4555/2014  ...............................................K.......................................R............
B/Thailand/CU-H3591/2014  ...............................................K.......................................R............
```

```
                                 110       120       130       140       150       160       170       180       190       200
                          ....|....|....|....|....|....|....|....|....|....|....|....|....|....|....|....|....|....|....|....|
B/Florida/4/2006          RTKIRQLPNLLRGYENIRLSTQNVIDAEKAPGGPYRLGTSGSCPNATSKSGFFATMAWAVPKDNNKNATNPLTVEVPYICTEGEDQITVWGFHSDDKTQM
B/Wisconsin/1/2010        .................................................I..............Y...................................
B/Bangladesh/3333/2007    .................................................I..............Y...................................
B/Stockholm/12/2011       .................................................I..............Y......Q............................
B/Thailand/CU-H2933/2011  .................................................I..............Y..............................N....
B/Thailand/CU-B3313/2012  ...............K.................................I..............Y..............................N....
B/Thailand/CU-H3496/2012  ...............K.................................I..............Y..............................N....
B/Thailand/CU-B6096/2012  .................................................I..............Y......Q.......................N....
B/Massachusetts/2/2012    .......A........................................................................A...................
B/Estonia/55669/2011      .......A........................................................................A...................
B/Thailand/CU-H3349/2012  .......A........................................................................A..............N....
B/Thailand/CU-H3456/2012  .......A............A...........................................................A..............N....
B/Thailand/CU-B6078/2011  .......A........................................................................A..............N....
B/Thailand/CU-A585/2013   .......A........................................................................A..............N....
B/Thailand/CU-B8813/2013  .......A........................................................................A..............N....
B/Thailand/CU-B8332/2013  .......A......................................A.................................A..............N....
B/Thailand/CU-A605/2014   .......A........................................................................A..............N....
B/Thailand/CU-A615/2014   .......A........................................................................A..............N....
B/Thailand/CU-A626/2014   .......A........................................................................A..............N....
B/Thailand/CU-A645/2014   .......A........................................................................A..............N....
B/Thailand/CU-B8925/2014  .......A........................................................................A..............N....
B/Thailand/CU-B8999/2014  .......A........................................................................A..............N....
B/Thailand/CU-B9017/2014  .......A........................................................................A..............N....
B/Thailand/CU-B9034/2014  .......A........................................................................A..............N....
B/Thailand/CU-C4555/2014  .......A........................................................................A..............N....
B/Thailand/CU-H3591/2014  .......A........................................................................A..............N....
```

```
                                 210       220       230       240       250       260       270       280       290       300
                          ....|....|....|....|....|....|....|....|....|....|....|....|....|....|....|....|....|....|....|....|
B/Florida/4/2006          KNLYGDSNPQKFTSSANGVTTHYVSQIGSFPDQTEDGGLPQSGRIVVDYMMQKPGKTGTIVYQRGVLLPQKVWCASGRSKVIKGSLPLIGEADCLHEKYG
B/Wisconsin/1/2010        .S..........................D.......................................................................
B/Bangladesh/3333/2007    ............................D.......................................................................
B/Stockholm/12/2011       ............................D.....................V.................................................
B/Thailand/CU-H2933/2011  ............................D.......................................................................
B/Thailand/CU-B3313/2012  .S..........................D..........................R.........................................E..
B/Thailand/CU-H3496/2012  .S..........................D..........................R.........................................E..
B/Thailand/CU-B6096/2012  ............................D.....................V.................................................
B/Massachusetts/2/2012    ............................G.......................................................................
B/Estonia/55669/2011      ............................G.......................................................................
B/Thailand/CU-H3349/2012  ............................G.......................................................................
B/Thailand/CU-H3456/2012  ..................I.........G.........................R.............................................
B/Thailand/CU-B6078/2011  ............................G.......................................................................
B/Thailand/CU-A585/2013   ............................G....R..................................................................
B/Thailand/CU-B8813/2013  ............................G....R..................................................................
B/Thailand/CU-B8332/2013  ............................G.......................................................................
B/Thailand/CU-A605/2014   ............................G.......................................................................
B/Thailand/CU-A615/2014   ............................G....R..................................................................
B/Thailand/CU-A626/2014   ............................G....R..................................................................
B/Thailand/CU-A645/2014   ............................G....R..................................................................
B/Thailand/CU-B8925/2014  ............................G....R..................................................................
B/Thailand/CU-B8999/2014  ............................G....R..................................................................
B/Thailand/CU-B9017/2014  ............................G....R....................E.............................................
B/Thailand/CU-B9034/2014  ............................G....R..................................................................
B/Thailand/CU-C4555/2014  ............................G....R..................................................................
B/Thailand/CU-H3591/2014  ..........E.................G....R..................................................................
```

```
                               310       320       330       340     HA2         360       370       380       390       400
                          ....|....|....|....|....|....|....|....|....|....|....|....|....|....|....|....|....|....|....|....|
B/Florida/4/2006          GLNKSKPYYTGEHAKAIGNCPIWVKTPLKLANGTKYRPPAKLLKERGFFGAIAGFLEGGWEGMIAGWHGYTSHGAHGVAVAADLKSTQEAINKITKNLNS
B/Wisconsin/1/2010        ....................................................................................................
B/Bangladesh/3333/2007    ....................................................................................................
B/Stockholm/12/2011       ....................................................................................................
B/Thailand/CU-H2933/2011  ....................................................................................................
B/Thailand/CU-B3313/2012  ...........K........................................................................................
B/Thailand/CU-H3496/2012  ...........K........................................................................................
B/Thailand/CU-B6096/2012  ....................................................................................................
B/Massachusetts/2/2012    ....................................................................................................
B/Estonia/55669/2011      ....................................................................................................
B/Thailand/CU-H3349/2012  ....................................................................................................
B/Thailand/CU-H3456/2012  ....................................................................................................
B/Thailand/CU-B6078/2011  ....................................................................................................
B/Thailand/CU-A585/2013   ....................................................................................................
B/Thailand/CU-B8813/2013  ....................................................................................................
B/Thailand/CU-B8332/2013  ............................E.......................................................................
B/Thailand/CU-A605/2014   ....................................................................................................
B/Thailand/CU-A615/2014   ....................................................................................................
B/Thailand/CU-A626/2014   ....................................................................................................
B/Thailand/CU-A645/2014   ....................................................................................................
B/Thailand/CU-B8925/2014  ....................................................................................................
B/Thailand/CU-B8999/2014  ....................................................................................................
B/Thailand/CU-B9017/2014  ....................................................................................................
B/Thailand/CU-B9034/2014  ....................................................................................................
B/Thailand/CU-C4555/2014  ....................................................................................................
B/Thailand/CU-H3591/2014  ....................................................................................................
```

```
                               410       420       430       440       450       460       470       480       490       500
                          ....|....|....|....|....|....|....|....|....|....|....|....|....|....|....|....|....|....|....|....|
B/Florida/4/2006          LSELEVKNLQRLSGAMDELHNEILELDEKVDDLRADTISSQIELAVLLSNEGIINSEDEHLLALERKLKKMLGPSAVEIGNGCFETKHKCNQTCLDRIAA
B/Wisconsin/1/2010        .............................................................................D......................
B/Bangladesh/3333/2007    .............................................................................D......................
B/Stockholm/12/2011       .............................................................................D......................
B/Thailand/CU-H2933/2011  .............................................................................D......................
B/Thailand/CU-B3313/2012  .............................................................................D......................
B/Thailand/CU-H3496/2012  .............................................................................D......................
B/Thailand/CU-B6096/2012  .............................................................................D......................
B/Massachusetts/2/2012    .............................................................................D......................
B/Estonia/55669/2011      .............................................................................D......................
B/Thailand/CU-H3349/2012  .............................................................................D......................
B/Thailand/CU-H3456/2012  .............................................................................D......................
B/Thailand/CU-B6078/2011  .............................................................................D......................
B/Thailand/CU-A585/2013   .............................................................................D......................
B/Thailand/CU-B8813/2013  .............................................................................D......................
B/Thailand/CU-B8332/2013  .............................................................................D......................
B/Thailand/CU-A605/2014   .............................................................................D......................
B/Thailand/CU-A615/2014   .............................................................................D......................
B/Thailand/CU-A626/2014   .............................................................................D......................
B/Thailand/CU-A645/2014   .............................................................................D......................
B/Thailand/CU-B8925/2014  .............................................................................D......................
B/Thailand/CU-B8999/2014  .............................................................................D......................
B/Thailand/CU-B9017/2014  .............................................................................D......................
B/Thailand/CU-B9034/2014  .............................................................................D......................
B/Thailand/CU-C4555/2014  .............................................................................D......................
B/Thailand/CU-H3591/2014  .............................................................................D......................
```

```
                               510       520       530       540       550       560       570
                          ....|....|....|....|....|....|....|....|....|....|....|....|....|....|
B/Florida/4/2006          GTFNAGEFSLPTFDSLNITAASLNDDGLDNHTILLYYSTAASSLAVTLMLAIFIVYMVSRDNVSCSICL*
B/Wisconsin/1/2010        .....................................................................*
B/Bangladesh/3333/2007    .....................................................................*
B/Stockholm/12/2011       .....................................................................*
B/Thailand/CU-H2933/2011  .....................................................................*
B/Thailand/CU-B3313/2012  .....................................................................*
B/Thailand/CU-H3496/2012  .....................................................................*
B/Thailand/CU-B6096/2012  .....................................................................*
B/Massachusetts/2/2012    .....................................................................*
B/Estonia/55669/2011      .....................................................................*
B/Thailand/CU-H3349/2012  .....................................................................*
B/Thailand/CU-H3456/2012  ...........................................................K.........*
B/Thailand/CU-B6078/2011  .....................................................................*
B/Thailand/CU-A585/2013   .....................................................................*
B/Thailand/CU-B8813/2013  .....................................................................*
B/Thailand/CU-B8332/2013  .....................................................................*
B/Thailand/CU-A605/2014   .....................................................................*
B/Thailand/CU-A615/2014   .....................................................................*
B/Thailand/CU-A626/2014   .....................................................................*
B/Thailand/CU-A645/2014   .....................................................................*
B/Thailand/CU-B8925/2014  .....................................................................*
B/Thailand/CU-B8999/2014  .....................................................................*
B/Thailand/CU-B9017/2014  .....................................................................*
B/Thailand/CU-B9034/2014  .....................................................................*
B/Thailand/CU-C4555/2014  .....................................................................*
B/Thailand/CU-H3591/2014  .....................................................................*
```

# NA protein alignment for influenza B virus isolated in Thailand, 2010 – 2014

Reference strains, Vaccine strains, Thailand strains; Potential N-linked glycosylation sites; Catalytic site and framework residues

```
                                   10        20        30        40        50        60        70        80        90       100
                             ....|....|....|....|....|....|....|....|....|....|....|....|....|....|....|....|....|....|....|....|
B/Florida/4/2006             MLPSTIQTLTLFLTSGGVLLSLYVSASLSYLLYSDILLKFSQTEITAPIMPLDCANASNVQAVNRSAAKGVTLLLPEPEWTYPRLSCPGSTFQKALLISP
B/Perth/211/2001             .........................................P......T..................T................................
B/Thailand/CU-B6078/2012     ................................................................H...................................
B/Thailand/CU-H3349/2012     .................M..............................................H.............K....................
B/Thailand/CU-H3456/2012     ....................................................................................................
B/Thailand/CU-A585/2013      ....................................................................................................
B/Thailand/CU-B8813/2013     ....................................................................................................
B/Thailand/CU-H8332/2013     .......................A........................................H....R..............................
B/Thailand/CU-A605/2014      .......................M.........................I..................................................
B/Thailand/CU-A615/2014      .........................................K..........................................................
B/Thailand/CU-A626/2014      .........................................K..........................................................
B/Thailand/CU-A645/2014      ................................................................H...................................
B/Thailand/CU-B8925/2014     ....................................................................................................
B/Thailand/CU-B8999/2014     ....................................................................................................
B/Thailand/CU-B9017/2014     ....................................................................................................
B/Thailand/CU-B9034/2014     ....................................................................................................
B/Thailand/CU-C4555/2014     .........................................K..........................................................
B/Thailand/CU-H3591/2014     ....................................................................................................
B/Wisconsin/1/2010           .........................................R.........................T................................
B/Thailand/CU-H2933/2011     .........................................R..V......................T....F...........................
B/Thailand/CU-B6096/2012     .........................................R.........................T...IP...........................
B/Thailand/CU-H3313/2012     .........................................R.........................T................................
B/Thailand/CU-H3496/2012     ...................................M.....R.........................T................................
B/Malaysia/2506/2004         ........................................PS......T..................T................................
B/Thailand/CU-243/2006       .........................................P......T..................T................................
B/Thailand/CU-364/2008       .........................................P......T..................T................................
B/Brisbane/60/2008           .........................................P......T..................T................................
B/Thailand/CU-B2271/2010     ..................................G......P......T..................T......................I.........
B/Thailand/CU-B2660/2010     ..................................G......P......T...............L..T......................I.........
B/Thailand/CU-B3153/2010     .........................................P......T..................T................................
B/Thailand/CU-C1262/2010     .........................................P......T..................T................................
B/Thailand/CU-C1451/2010     .........................................P......T..................T................................
B/Thailand/CU-B2504/2010     .........................................P......T..................T................................
B/Thailand/CU-B4505/2011     .........................................P......T..................T................................
B/Thailand/CU-B5522/2011     .........................................P......T..................T................................
B/Thailand/CU-B5734/2011     .........................................P......T..................T................................
B/Thailand/CU-C1768/2011     .........................................P......T..................T................................
B/Thailand/CU-H3002/2011     .........................................P......T..................T................................
B/Thailand/CU-H3052/2011     ....................L....................P......T..................T................................
B/Thailand/CU-B6148/2012     .........................................P......T...................................................
B/Thailand/CU-B7215/2012     .........................................P......T..................T................................
B/Thailand/CU-B6257/2012     .........................................P......T..................T....F...........................
B/Thailand/CU-B6975/2012     .........................................P..M...T..................T................................
B/Thailand/CU-B7337/2012     .........................................P......T..................T.......S........................
B/Thailand/CU-B2201/2010     .........................................P......T..................T...................Q............
B/Thailand/CU-B2320/2010     .........................................P......T..................T................................
B/Thailand/CU-B2372/2010     ..........................L..............P......T.S................T....F...........................
B/Thailand/CU-B2390/2010     .......M......L..........................PP.....T..................T................................
B/Thailand/CU-B2432/2010     .........................................P......TI.................T................................
B/Thailand/CU-H1400/2010     .........................................P......T..................T...................Q............
B/Thailand/CU-H1896/2010     .........................................P......T..................T................................
B/Thailand/CU-H2132/2010     .........................................P......T..................T................................
B/Thailand/CU-H2584/2010     .........................................P....T.T..................T................................
B/Thailand/CU-H2738/2010     ..........................L..............P......T.S................T....F...........................
B/Thailand/CU-B4585/2011     .......M......L..................L......PP......T..................T................................
B/Thailand/CU-B5671/2011     .......................L..L..............P......T.S................T....F...........................
B/Thailand/CU-B5910/2011     .........................................P......T..................T....F...........................
B/Thailand/CU-B6240/2012     .........................................P......T..................T................................
```

```
                                   110       120       130       140       150       160       170       180       190       200
                               ....|....|....|....|....|....|....|....|....|....|....|....|....|....|....|....|....|....|....|....|
B/Florida/4/2006               HRFGETKGNSAPLIIREPFIACGPTECKHFALTHYAAQPGGYYNGTREDRNKLRHLISVKLGKIPTVENSIFHMAAWSGSACHDGKEWTYIGVDGPDSNA
B/Perth/211/2001               ........................K......................G................................................EN..
B/Thailand/CU-B6078/2012       .....I...........................................................................................N..
B/Thailand/CU-H3349/2012       .....I..............................................................................................
B/Thailand/CU-H3456/2012       .....I.....................R........................................................................
B/Thailand/CU-A585/2013        .....I..............................................................................................
B/Thailand/CU-B8813/2013       .....I..............................................................................................
B/Thailand/CU-H8332/2013       .....I..............................................................................................
B/Thailand/CU-A605/2014        .....I..............................................................................................
B/Thailand/CU-A615/2014        .....I..............................................................................................
B/Thailand/CU-A626/2014        .....I..............................................................................................
B/Thailand/CU-A645/2014        .....I..............................................................................................
B/Thailand/CU-B8925/2014       .....I..............................................................................................
B/Thailand/CU-B8999/2014       .....I..............................................................................................
B/Thailand/CU-B9017/2014       .....I..............................................................................................
B/Thailand/CU-B9034/2014       .....I..............................................................................................
B/Thailand/CU-C4555/2014       .....I..............................................................................................
B/Thailand/CU-H3591/2014       .....I..............................................................................................
B/Wisconsin/1/2010             ........................K............................................................R..............
B/Thailand/CU-H2933/2011       ...................V....K............................................................R..............
B/Thailand/CU-B6096/2012       ........................K............................................................R..............
B/Thailand/CU-H3313/2012       ........................K............................................................R..............
B/Thailand/CU-H3496/2012       ........................K............................................................R..............
B/Malaysia/2506/2004           ........................K......................G.................................................N..
B/Thailand/CU-243/2006         ........................S......................G.................................................N..
B/Thailand/CU-364/2008         .....A..................S......................G.................................................N..
B/Brisbane/60/2008             ........................N......................G.................................................N..
B/Thailand/CU-B2271/2010       ........................N......................G.................................................N..
B/Thailand/CU-B2660/2010       ........................N......................G.................................................N..
B/Thailand/CU-B3153/2010       ........................N......................G.................................................N..
B/Thailand/CU-C1262/2010       ........................N......................G............................................I....N..
B/Thailand/CU-C1451/2010       ........................N......................G.................................................N..
B/Thailand/CU-B2504/2010       ........................N......................G.................................................N..
B/Thailand/CU-B4505/2011       ........................N......................G.................................................N..
B/Thailand/CU-B5522/2011       ........................N......................G.................................................N..
B/Thailand/CU-B5734/2011       ........................N......................G.................................................N..
B/Thailand/CU-C1768/2011       ........................N......................G.................................................N..
B/Thailand/CU-H3002/2011       ........................N......................G.................................................N..
B/Thailand/CU-H3052/2011       ........................N......................G.................................................N..
B/Thailand/CU-B6148/2012       ........................N......................G.................................................N..
B/Thailand/CU-B7215/2012       ........................N......................G.................................................N..
B/Thailand/CU-B6257/2012       ........................N......................G.................................................N..
B/Thailand/CU-B6975/2012       ........................N......................G.................................................N..
B/Thailand/CU-B7337/2012       ........................N......................G.................................................N..
B/Thailand/CU-B2201/2010       ........................N......................G.................................................N..
B/Thailand/CU-B2320/2010       ........................N......................G.................................................N..
B/Thailand/CU-B2372/2010       ........................N......................G.................................................ND.
B/Thailand/CU-B2390/2010       .....A..................K......................G.................................................N..
B/Thailand/CU-B2432/2010       ........................N......................G.................................................N..
B/Thailand/CU-H1400/2010       ........................N......................G.................................................N..
B/Thailand/CU-H1896/2010       ........................N......................G.................................................N..
B/Thailand/CU-H2132/2010       ........................N...............R......G.................................................N..
B/Thailand/CU-H2584/2010       ........................N......................G.................................................N..
B/Thailand/CU-H2738/2010       ........................N......................G.......................L.........................ND.
B/Thailand/CU-B4585/2011       ........................K......................G.................................................N..
B/Thailand/CU-B5671/2011       ........................N......................G.................................................ND.
B/Thailand/CU-B5910/2011       ........................N......................G.................................................N..
B/Thailand/CU-B6240/2012       ........................N......................G.................................................N..
```

```
                                  210       220       230       240       250       260       270       280       290       300
                          ....|....|....|....|....|....|....|....|....|....|....|....|....|....|....|....|....|....|....|....|
B/Florida/4/2006          LLKIKYGEAYTDTYHSYAKNILRTQESACNCIGGDCYLMITDGPASGISECRFLKIREGRIIKEIFPTGRVKHTEECTCGFASNKTIECACRDNSYTAKR
B/Perth/211/2001          ..................N...............N........S........................................................
B/Thailand/CU-B6078/2012  ...............................................V..............................................R.....
B/Thailand/CU-H3349/2012  ...............................................V..............................................R.....
B/Thailand/CU-H3456/2012  ...............................................V..............................................R.....
B/Thailand/CU-A585/2013   ...............................................V..............................................H.....
B/Thailand/CU-B8813/2013  ...............................................V..............................................R.....
B/Thailand/CU-H8332/2013  ...............................................V..............................................R.....
B/Thailand/CU-A605/2014   ................................R..............V..............................................R.....
B/Thailand/CU-A615/2014   ...............................................V..............................................R.....
B/Thailand/CU-A626/2014   ...............................................V..............................................R.....
B/Thailand/CU-A645/2014   ...............................................V..............................................R.....
B/Thailand/CU-B8925/2014  ...............................................V..............................................R.....
B/Thailand/CU-B8999/2014  ...............................................V..............................................R.....
B/Thailand/CU-B9017/2014  ...............................................V..............................................R.....
B/Thailand/CU-B9034/2014  ...............................................V..............................................H.....
B/Thailand/CU-C4555/2014  ...............................................V..............................................R.....
B/Thailand/CU-H3591/2014  ...............................................V..............................................R.....
B/Wisconsin/1/2010        ....................................................................................................
B/Thailand/CU-H2933/2011  ....................................................................................................
B/Thailand/CU-B6096/2012  ....................................................................................................
B/Thailand/CU-H3313/2012  ....................................................................................................
B/Thailand/CU-H3496/2012  ....................................................................................................
B/Malaysia/2506/2004      ..................N...............N........S...V......................I.............................
B/Thailand/CU-243/2006    ..................NK..............N........S...V....................................E...............
B/Thailand/CU-364/2008    ..................NK..............N........S...V....................................E...............
B/Brisbane/60/2008        ...V..............NK..............N........S...V....................................................
B/Thailand/CU-B2271/2010  ...V..............NK..............N........S...V....................................................
B/Thailand/CU-B2660/2010  ...V..............NK..............N........S...V....................................................
B/Thailand/CU-B3153/2010  ...V..............NK..............N........S...V....................................................
B/Thailand/CU-C1262/2010  ...V..............NK..............N........S...V....................................................
B/Thailand/CU-C1451/2010  ...V..............NK..............N........S...V....................................................
B/Thailand/CU-B2504/2010  ...V..............NK..............N........S...V....................................................
B/Thailand/CU-B4505/2011  ...V..............NK..............N........S...V....................................................
B/Thailand/CU-B5522/2011  ...V..............NK..............N........S...V....................................................
B/Thailand/CU-B5734/2011  ...V..............NK..............N........S...V....................................................
B/Thailand/CU-C1768/2011  ...V..............NK..............N........S...V....................................................
B/Thailand/CU-H3002/2011  ...V..............NK..............N........S...V....................................................
B/Thailand/CU-H3052/2011  ...V..............NK..............N........S...V....................................................
B/Thailand/CU-B6148/2012  ...V..............NK..............N........S...V....................................................
B/Thailand/CU-B7215/2012  ...V..............NK..............N........S...V....................................................
B/Thailand/CU-B6257/2012  ...V..............NK..............N........S...V..............................................R.....
B/Thailand/CU-B6975/2012  ...V..............NK..............N........S...V..............................................R.....
B/Thailand/CU-B7337/2012  ...V..............NK..............N........S...V..............................................R.....
B/Thailand/CU-B2201/2010  ...V..............NK..............N........S...V....................................................
B/Thailand/CU-B2320/2010  ...V..............NK..............N........S...V....................................................
B/Thailand/CU-B2372/2010  ...V..............NK..............N........S...V....................................................
B/Thailand/CU-B2390/2010  ..................N...............N........S...V....................................................
B/Thailand/CU-B2432/2010  ...V..............NK..............N........S...V....................................................
B/Thailand/CU-H1400/2010  ...V..............NK..............N........S...V....................................................
B/Thailand/CU-H1896/2010  ...V..............NK..............N........S...V....................................................
B/Thailand/CU-H2132/2010  ...V..............NK..............N........S...V....................................................
B/Thailand/CU-H2584/2010  ...V..............NK..............N........S...V....................................................
B/Thailand/CU-H2738/2010  ...V..............NK..............N........S...V....................................................
B/Thailand/CU-B4585/2011  .........F........N...............N........S...V....................................................
B/Thailand/CU-B5671/2011  ...V..............NK............E.N........S...V.............V......................................
B/Thailand/CU-B5910/2011  ...V..............NE..............N........S...V....................................................
B/Thailand/CU-B6240/2012  ..................NK..............N........S...V..............................................R.....
```

```
                             310       320       330       340       350       360       370       380       390       400
                         ....|....|....|....|....|....|....|....|....|....|....|....|....|....|....|....|....|....|....|....|
B/Florida/4/2006         PFVKLNVETDTAEIRLMCTETYLDTPRPNDGSITGPCESDGDKGSGGIKGGFVHQRMASKIGRWYSRTMSKTKRMGMGLYVKYDGDPWTDSEALALSGVM
B/Perth/211/2001         ............................D..........N...................................................D........
B/Thailand/CU-B6078/2012 ....................................................................................................
B/Thailand/CU-H3349/2012 ...........................................................................................G........
B/Thailand/CU-H3456/2012 .........................................................T..........................................
B/Thailand/CU-A585/2013  ............K.............................................F.........................................
B/Thailand/CU-B8813/2013 ....................................................................................................
B/Thailand/CU-H8332/2013 ....................................................................................................
B/Thailand/CU-A605/2014  ....................................................................................................
B/Thailand/CU-A615/2014  ............................................N.......................................................
B/Thailand/CU-A626/2014  ....................................................................................................
B/Thailand/CU-A645/2014  ....................................................................................................
B/Thailand/CU-B8925/2014 ....................................................................................................
B/Thailand/CU-B8999/2014 ....................................................................................................
B/Thailand/CU-B9017/2014 ....................................................................................................
B/Thailand/CU-B9034/2014 ....................................................................................................
B/Thailand/CU-C4555/2014 ....................................................................................................
B/Thailand/CU-H3591/2014 ....................................................................................................
B/Wisconsin/1/2010       .......................................N............................................................
B/Thailand/CU-H2933/2011 .......................................N............................................................
B/Thailand/CU-B6096/2012 ...................K...................N..E.........................................................
B/Thailand/CU-H3313/2012 .......................................N............................................................
B/Thailand/CU-H3496/2012 .......................................N............................................................
B/Malaysia/2506/2004     ............................D..........N................................................A..D...F....
B/Thailand/CU-243/2006   ...................D........D..........N................................E...............A..D...F....
B/Thailand/CU-364/2008   ...................D........D..........N................................E...............A..D...F....
B/Brisbane/60/2008       ...................D...................N.................E..............E...............A..D...F....
B/Thailand/CU-B2271/2010 ...................D...................N.................E..............E...............A..D...F....
B/Thailand/CU-B2660/2010 ...................D...................N.................E..............E...............A..D...F....
B/Thailand/CU-B3153/2010 ...................D...................N.................E..............E...............A..D...F....
B/Thailand/CU-C1262/2010 ...................D...................N.................E..............E...............A..D...F....
B/Thailand/CU-C1451/2010 ...................D...................N.................E..............E...............A..D...F....
B/Thailand/CU-B2504/2010 ...................D...................N.................E..............E...............A..D...F....
B/Thailand/CU-B4505/2011 ...................D...................N.................E..............E...............A..D...F....
B/Thailand/CU-B5522/2011 ...................D...................N.................E..............E....E..........A..D..DF....
B/Thailand/CU-B5734/2011 ...................D...................N.................E..............E....E..........A..D..DF....
B/Thailand/CU-C1768/2011 ...................D...................N.................E..............E...............A..D...F....
B/Thailand/CU-H3002/2011 ...................D...................N.................E..............E....E..........A..D..DF....
B/Thailand/CU-H3052/2011 ...................D...................N.................E..............E...............A..D...F....
B/Thailand/CU-B6148/2012 ...................D...................N.E...............E..............E...............A..D..TF....
B/Thailand/CU-B7215/2012 ...................D...................N.................E..............E....E..........A..D..EF....
B/Thailand/CU-B6257/2012 ...................D.....................................K..............E...............A..D...F....
B/Thailand/CU-B6975/2012 ...................D.....................................K..............E...............A..D...F..A.
B/Thailand/CU-B7337/2012 ...................D........................N............K..............E...............A..D...F....
B/Thailand/CU-B2201/2010 ...................D........D..........N.................E..............E...............A..D..TF....
B/Thailand/CU-B2320/2010 ...................D........D..........N.................E..............E...............A..D..TF....
B/Thailand/CU-B2372/2010 ..I................D........D..........N.................E..............E...............A..D...F....
B/Thailand/CU-B2390/2010 ............................D..........N................................E...............A..D...F....
B/Thailand/CU-B2432/2010 ...................D........D..........N.................E..............E...............A..D..TF....
B/Thailand/CU-H1400/2010 ...................D........D..........N.................E..............E...............A..D..TF....
B/Thailand/CU-H1896/2010 ...................D........D..........N.................E..............E...............A..D..TF....
B/Thailand/CU-H2132/2010 ...................D........D..........N.................E..............E...............A..D..TF....
B/Thailand/CU-H2584/2010 ...................D........D..........N.................E..............E...............AE.D..TF....
B/Thailand/CU-H2738/2010 ...................D........D..........N.................E..............E...............A..D...F....
B/Thailand/CU-B4585/2011 ............................D..........N................................E...............A..D...F....
B/Thailand/CU-B5671/2011 ...................D........D..........N.................K..............E...............A..D...F....
B/Thailand/CU-B5910/2011 ...................D........D..........N.................E..............E...............A..D..TF....
B/Thailand/CU-B6240/2012 ...................D.....................................K..............E...............A..D...F....
```

```
                                    410       420       430       440       450       460
                            ....|....|....|....|....|....|....|....|....|....|....|....|..
B/Florida/4/2006            VSMEEPGWYSFGFEIKDKKCDVPCIGIEMVHDGGKTTWHSAATAIYCLMGSGQLLWDTVTGVDMAL*
B/Perth/211/2001            ...................................E..............................*
B/Thailand/CU-B6078/2012    ..................................................................*
B/Thailand/CU-H3349/2012    ..................................................................*
B/Thailand/CU-H3456/2012    ..................................................................*
B/Thailand/CU-A585/2013     ..................................................................*
B/Thailand/CU-B8813/2013    ..................................................................*
B/Thailand/CU-H8332/2013    ..................................................................*
B/Thailand/CU-A605/2014     ..................................................................*
B/Thailand/CU-A615/2014     ...........................................S......................*
B/Thailand/CU-A626/2014     ..................................................................*
B/Thailand/CU-A645/2014     ..................................................................*
B/Thailand/CU-B8925/2014    ..................................................................*
B/Thailand/CU-B8999/2014    ..................................................................*
B/Thailand/CU-B9017/2014    ..................................................................*
B/Thailand/CU-B9034/2014    ..................................................................*
B/Thailand/CU-C4555/2014    ..................................................................*
B/Thailand/CU-H3591/2014    ..................................................................*
B/Wisconsin/1/2010          ..............................................................N.T.*
B/Thailand/CU-H2933/2011    ..I...........................................................N.T.*
B/Thailand/CU-B6096/2012    ..............................................................N.T.*
B/Thailand/CU-H3313/2012    ..............................................................N.T.*
B/Thailand/CU-H3496/2012    ..............................................................N.T.*
B/Malaysia/2506/2004        ...................................E..........................N...*
B/Thailand/CU-243/2006      ...K...............................E..............................*
B/Thailand/CU-364/2008      ...K...............................E..............................*
B/Brisbane/60/2008          ...K...............................E..............................*
B/Thailand/CU-B2271/2010    ...K...............................E..............................*
B/Thailand/CU-B2660/2010    ...K...............................E..............................*
B/Thailand/CU-B3153/2010    ...K...............................E..............................*
B/Thailand/CU-C1262/2010    ...K...............................E..............................*
B/Thailand/CU-C1451/2010    ...K...............................E..............................*
B/Thailand/CU-B2504/2010    ...K...............................E..............................*
B/Thailand/CU-B4505/2011    ...K...............................E..............................*
B/Thailand/CU-B5522/2011    ...K...............................E..........................N...*
B/Thailand/CU-B5734/2011    ...K...............................E..........................N...*
B/Thailand/CU-C1768/2011    ...K...............................E..............................*
B/Thailand/CU-H3002/2011    ...K...............................E..........................N...*
B/Thailand/CU-H3052/2011    ...K...............................E..............................*
B/Thailand/CU-B6148/2012    ...K...............................E..............................*
B/Thailand/CU-B7215/2012    ...K...............................E..........................N...*
B/Thailand/CU-B6257/2012    I..K...............................E..............................*
B/Thailand/CU-B6975/2012    ...K...............................E..............................*
B/Thailand/CU-B7337/2012    ...K...............................E..............................*
B/Thailand/CU-B2201/2010    ...K...............................E..............................*
B/Thailand/CU-B2320/2010    ...K...............................E..............................*
B/Thailand/CU-B2372/2010    ...K...............................E..............................*
B/Thailand/CU-B2390/2010    ...K...............................E..........................N...*
B/Thailand/CU-B2432/2010    ...K...............................E..............................*
B/Thailand/CU-H1400/2010    ...K...............................E..............................*
B/Thailand/CU-H1896/2010    ...K...............................E..............................*
B/Thailand/CU-H2132/2010    ...K...............................E..............................*
B/Thailand/CU-H2584/2010    ...K...............................E..............................*
B/Thailand/CU-H2738/2010    ...K...............................E..............................*
B/Thailand/CU-B4585/2011    ...K...............................E..........................N...*
B/Thailand/CU-B5671/2011    ...K...............................E..............................*
B/Thailand/CU-B5910/2011    ...K...............................E..............................*
B/Thailand/CU-B6240/2012    ...K...............................E..............................*
```
