# Supplementary material for: Molecular Epidemiology and Phylogenetic Analyses of Influenza B Virus in Thailand during 2010 to 2014
Source: PLoS One. 2015 Jan 20;10(1):e0116302. doi: 10.1371/journal.pone.0116302 (PMC4300180; doi:10.1371/journal.pone.0116302)
Supplement: S1 Table — (DOCX) [file pone.0116302.s008.docx]

**Table S1: Primer sets used for conventional PCR amplification of the whole genome of circulating Thailand influenza B strains**

| **Primer Name** | **Nucleotide Sequences (5’-3’)** | **PCR product (bp)** |
| --- | --- | --- |
| PB2-F5' | AGC AGA AGC GGA GCC TTT TCA AG | 1082 |
| PB2-R1082 | CCG TCC CAT ATT CCA ATC TTC TG |  |
| PB2-F902 | CAG CCA TAG ACG GAG GTG ATG T | 1005 |
| PB2-R1907 | TGA TAA GGC TCC CCA TTG CTC CTT |  |
| PB2-F1716 | GTT CCA ATG GGA TGC ATT TGA AG | 584 |
| PB2-R3 | ATG ACC AGT AGA AAC ACG AGC A |  |
| PB1-F5' | AGC AGA AGC GGA GCC TTT AAG ATG | 1024 |
| PB1-R1024 | CCG GTG CTA TAC TAC AAA AAT CCC |  |
| PB1-F896 | GCA TGA CAG TAA CAG GAG ACA AT | 1050 |
| PB1-R1946 | CCA TGT GCT GGG GTT ATA TCT GC |  |
| PB1-F1667 | ACA CCT ACA AAT GCC ACA GGG GAG | 639 |
| PB1-R3' | TGA CCA GTA GAA ACA CGA GCC TTT |  |
| PA-F5' | AGC AGA AGC GGT GCG TTT GAT TTG | 1114 |
| PA-R1114 | ATG TTA ATC CAT CCC CTG TGG CCC |  |
| PA-F1010 | GGA AGC TTT GGA GAG ACT GTG TAA A | 826 |
| PA-R1836 | GGG GCT ATT TAC TCT GTC TCC C |  |
| PA-F1622 | TTG GCT CCC TAT TTG TGA GTG GG | 578 |
| PA-R3' | AGT AGA AAC ACG TGC ATT TTT GAT TC |  |
| HA-F5' | AGC AGA AGC AGA GCA TTT TCT AA | 1142 |
| HA-R1142 | GCA ATC ATT CCT TCC CAT CCT CC |  |
| HA-F997 | ATA GGA AAT TGC CCA ATA TGG GT | 703 |
| HA-R3' | CGT TTC TTT GTA ATG ATG ACA AG |  |
| NP-F5' | AGC AGA AGT ACA GCA TTT TCT TG | 916 |
| NP-R916 | TCT TCA ATG TCT GCA ATC CCT GG |  |
| NP-F786 | GGC AGA CAG AGG GCT ATT GAG AG | 779 |
| NP-R3' | AGT AGA AAC AAC AGC ATT TTT TA |  |
| NA-F5' | AGC AGA AGC AGA GCA TCT TCT CAA A | 1165 |
| NA-R1165 | TTA GAC ATC GTT CGA GAG TAC CA |  |
| NA-F1014 | ACT TAT TTG GAC ACC CCC AGA CC | 386 |
| NA-R3' | AGT AGT AAC AAG AGC ATT TTT CAG A |  |
| M-F5' | ACC AGA AGC ACG CAC TTT CTT AA | 1000 |
| M-R3' | TGA CCA GTA GAA ACA ACG CAC |  |
| NS-F5' | AGC AGA AGC AGA GGA TTT GTT TAG | 900 |
| NS-R3' | ATG ACC AGT AGT AAC AAG AGG ATT |  |
