# Supplementary material for: Molecular Epidemiology and Phylogenetic Analyses of Influenza B Virus in Thailand during 2010 to 2014
Source: PLoS One. 2015 Jan 20;10(1):e0116302. doi: 10.1371/journal.pone.0116302 (PMC4300180; doi:10.1371/journal.pone.0116302)
Supplement: S2 Table — (DOCX) [file pone.0116302.s009.docx]

**Table S2: Accession numbers in GenBank and GISAID of eight influenza B gene sequences used to construct phylogenetic trees in this study.**

| **Gene** | **Accession No.** |
| --- | --- |
| **PB1** | CY018763 CY018771 CY019537 CY040455 CY115189  CY153888 CY171829 CY176319 CY156664 CY115157  CY115349 CY155704 CY115253 CY119944 CY033882  EF626642 EU305611 AJ781186 X266881 DQ792895 |
| **PB2** | CY018764 CY018772 CY019538 CY115198 CY115390  CY171854 CY175504 CY176128 CY175856 CY040456  CY033883 CY115158 CY115190 DQ792894 EF626643  FJ461688 EU305612 JX266889 AJ781207 |
| **PA** | CY018762 CY018770 CY019536 CY172132 CY171964  CY176358 CY153551 CY153887 CY155887 CY115404  CY040454 CY033881 CY115156 CY115188 DQ792896  EF626641 JX266897 KJ532166 |
| **HA** | AFH58304 AFH58348 AJ784040 AJ784056 AY504602  AGX18589 CY019675 CY019611 CY022221 DQ792897  CY018757 CY018765 CY019531 CY019619 CY153730  CY154530 CY022221 CY018677 CY156938 CY018453  CY115383 CY171959 CY173809 CY040449 CY033876  CY115151 CY115183 CY033844 CY149981 CY040441  M10298 KC891793 KC813773 KC892118 EPI406272  EPI171429 EPI366580 EPI193056 EPI301334 EPI340834  EPI271913 EPI406983 EU305614 EF626636 EPI 211559 |
| **NP** | CY018760 CY018768 CY019534 CY175092 CY171786  CY172050 CY153693 CY155901 CY115282 CY175852  CY040452 CY033879 CY115154 CY115186 DQ792898  EF626639 KJ532157 JX266940 |
| **NA** | CY018759 CY018767 CY019533 CY018471 CY018639  CY018343 CY115385 CY030777 CY018431 CY018351  CY171849 CY176291 CY150172 CY040451 CY033878  CY115153 CY115185 CY019677 CY019613 CY022223  DQ792899 EF626638 EF541477 JX266945 KF234468  KC478983 |
| **M** | CY018758 CY018766 CY019532 CY153275 CY153443  CY033941 CY153315 CY172096 CY176322 CY155899  CY115176 CY040450 CY033877 CY115152 CY115184  CY019676 CY019612 CY022222 DQ792900 EF626637  JX266954 JX266956 KC813859 AB120273 |
| **NS** | CY018761 CY018769 CY019535 CY171963 CY153886  CY033872 CY173797 CY153782 CY040453 CY033880  CY115155 CY115187 DQ792901 EF626640 KC891879  KC891927 KC892030 KJ532186 |
